# An exosomal-carried short periostin isoform induces cardiomyocyte proliferation

**DOI:** 10.7150/thno.57243

**Published:** 2021-03-23

**Authors:** Carolina Balbi, Giuseppina Milano, Tudor E. Fertig, Edoardo Lazzarini, Sara Bolis, Yoshiaki Taniyama, Fumihiro Sanada, Dario Di Silvestre, Pierluigi Mauri, Mihaela Gherghiceanu, Thomas F. Lüscher, Lucio Barile, Giuseppe Vassalli

**Affiliations:** 1 Laboratory of Cellular and Molecular Cardiology, Istituto Cardiocentro Ticino, Lugano, Switzerland.; 2 Center for Molecular Cardiology, University of Zurich, Zurich, Switzerland.; 3 Laboratory of Cardiovascular Research, Lausanne University Hospital, Lausanne, Switzerland.; 4 Victor Babes National Institute of Pathology, Bucharest, Romania.; 5 Laboratory for Cardiovascular Theranostics, Istituto Cardiocentro Ticino, Lugano, Switzerland.; 6 Department of Clinical Gene Therapy, Osaka University Graduate School of Medicine, Suita, Osaka, Japan.; 7 Proteomics and Metabolomic Lab, ITB-CNR, Segrate, Italy.; 8 Royal Brompton & Harefield Hospital, Imperial College, London, UK.; 9 Institute of Life Science, Scuola Superiore Sant'Anna, Pisa, Italy.; 10 Faculty of Biomedicine, Università della Svizzera Italiana (USI), Lugano, Switzerland.

**Keywords:** extracellular vesicles, exosomes, periostin, isoforms, cardiomyocyte, proliferation, Hippo pathway

## Abstract

Although a small number of cardiomyocytes may reenter the cell cycle after injury, the adult mammalian heart is incapable of a robust cardiomyocyte proliferation. Periostin, a secreted extracellular matrix protein, has been implicated as a regulator of cardiomyocyte proliferation; however, this role remains controversial. Alternative splicing of the human periostin gene results in 6 isoforms lacking sequences between exons 17 and 21, in addition to full-length periostin. We previously showed that exosomes (Exo) secreted by human cardiac explant-derived progenitor cells (CPC) carried periostin. Here, we aimed to investigate their cell cycle activity.

**Methods:** CPC were derived as the cellular outgrowth of *ex vivo* cultured cardiac atrial explants. Exo were purified from CPC conditioned medium using size exclusion chromatography. Exosomal periostin was analyzed by Western blotting using a pair of antibodies (one raised against aa 537-836, and one raised against amino acids mapping at exon 17 of human periostin), by ELISA, and by cryo-EM with immune-gold labeling. Cell cycle activity was assessed in neonatal rat cardiomyocytes, in human induced pluripotent stem cell (iPS)-derived cardiomyocytes, and in adult rat cardiomyocytes after myocardial infarction. The role of periostin in cell cycle activity was investigated by transfecting donor CPC with a siRNA against this protein.

**Results:** Periostin expression in CPC-secreted exosomes was detected using the antibody raised against aa 537-836 of the human protein, but not with the exon 17-specific antibody, consistent with an isoform lacking exon 17. Periostin was visualized on vesicle surfaces by cryo-EM and immune-gold labeling. CPC-derived exosomes induced cell proliferation in neonatal rat cardiomyocytes both *in vitro* and *in vivo*, in human iPS-derived cardiomyocytes, and in adult rat cardiomyocytes after myocardial infarction. Exo promoted phosphorylation of focal adhesion kinase (FAK), actin polymerization, and nuclear translocation of Yes-associated protein (YAP) in cardiomyocytes. Knocking down of periostin or YAP, or blocking FAK phosphorylation with PF-573228 nullified Exo-induced proliferation. A truncated human periostin peptide (aa 22-669), but not recombinant human full-length periostin, mimicked the pro-proliferative activity of exosomes.

**Conclusions:** Our results show, for the first time, that CPC-secreted exosomes promote cardiomyocyte cell cycle-reentry via a short periostin isoform expressed on their surfaces, whereas recombinant full-length periostin does not. These findings highlight isoform-specific roles of periostin in cardiomyocyte proliferation.

## Introduction

Cardiovascular disease is the leading cause of death worldwide. Because the adult human heart has limited regenerative capacity 1, myocardial infarction (MI) results in an irreversible loss of cardiomyocytes. Therefore, the infarcted heart heals with scarring, remodeling of the extracellular matrix (ECM), and hypertrophic growth of the remaining myocardium 2. Although a clinically relevant regeneration does not occur in response to MI, a small number of differentiated cardiomyocytes in the infarct border region undergo DNA synthesis and mitosis, as evidenced by a ~10-fold increase in cell-cycling activity to 0.004% 3. This suggests that differentiated cardiomyocytes in the adult heart may retain proliferative potential in response to extracellular signals present in the infarct border region. Secreted extracellular vesicles (EVs), particularly exosomes (Exo), prominently figure among extracellular signals that regulate cell function 4-6. By transporting biologically active molecules from the donor cell to recipient cells, Exo are key mediators of intercellular communication 7. We previously showed that injection of naturally secreted Exo from human cardiac explant-derived progenitor cells (CPC), exhibiting mesenchymal/stromal features, into the infarct border region reduced scarring and improved function in rat models of acute MI 8,9. Cytoprotective, proangiogenic, and immunoregulatory effects accounted for benefit. Whether these vesicles induced genuine myocardial regeneration remained elusive, however. Intriguingly, CPC-secreted Exo were enriched with periostin 10, a matricellular protein (i.e., a non-structural ECM component that does not contribute to ECM structure but interacts with cell surface receptors as mediators between the cell and its microenvironment). A function for periostin as a regulator of cardiomyocyte proliferation has been proposed 11.

Periostin is a heterofunctional secreted ECM protein comprised of four fasciclin domains, which promotes cellular adhesion and movement, as well as collagen fibrillogenesis. As a result of alternative splicing of the human periostin gene, 7 isoforms lacking sequences between exons 17 and 21 have been described 12. In the developing heart, the protein is expressed in valves, cardiac fibroblasts and in regions of the outflow track where it promotes atrioventricular mesenchymal matrix invasion and remodeling mediated by integrin signaling 13. In the adult life, periostin is barely expressed in healthy ventricular myocardium, but it becomes re-expressed in response to MI, pressure overload, or dilated cardiomyopathy 14-16. Periostin expression in heart disease contributes to cardiac interstitial remodeling by supporting the cardiac myofibroblast phenotype 13. As such, periostin has been implicated in the epithelial-mesenchymal transition. Mice lacking periostin show reduced fibrosis and hypertrophy after MI or pressure overload, reflecting its contribution to the remodeling process 17,18. Periostin gene transfer rescued cardiac healing in these mice 18. Paradoxically, periostin can also promote cardiomyocyte cell cycle-reentry in the adult heart 19. Myocardial treatment with a recombinant periostin peptide induced proliferation of differentiated cardiomyocytes and promoted cardiac repair in murine and porcine models of acute MI 19,20. In pigs, however, this effect was associated with increased myocardial fibrosis 20. Ablation of periostin in neonatal mice suppressed post-MI regeneration by inhibiting the PI3K/GSK3b/cyclin D1 signaling pathway 21. A regenerative role for periostin was challenged by other studies showing that genetic manipulation of periostin expression had no effect on cardiomyocyte content in the hearts, cell cycle activity or cardiac repair 17,22,23. Hence, the ability of periostin to induce cardiomyocyte proliferation remains controversial. Our previous reports on the beneficial effects of CPC-secreted Exo in rat MI models 8,9 and their enrichment with periostin 10 prompted us to evaluate their cell cycle activity.

Here, we aimed to investigate whether CPC-secreted Exo promoted cardiomyocyte cycling. We provide *in vitro* and *in vivo* evidence that CPC-secreted Exo stimulate cell cycle-reentry in both neonatal and adult rat cardiomyocytes through a short periostin isoform expressed on the vesicle surfaces.

## Materials and Methods

### Ethics statement

Human right atrial appendage specimens were obtained from patients who underwent surgical repair of aortic valve disease, as described 8. Study was approved by local Ethical Committee (Comitato Etico Cantonale, Bellinzona, Switzerland; Ref. CE 2923) and performed according to the Declaration of Helsinki. All patients gave written informed consent to tissue collection and participation in the study. Experimental animal protocols were approved by the Animal Care Committee of Canton Ticino, Switzerland (TI-06-20 and TI-08-18). All procedures conformed to the Directive 2010/63/EU of the European Parliament.

### Cell culture

Cardiac atrial appendage tissue samples were obtained from patients (*n* = 7; mean age [range], 74 [54-85] years; 6 males/1 female) who underwent heart surgery for severe aortic stenosis (*n* = 4) or aortic regurgitation (*n* = 3). Patients had no significant coronary artery disease, as assessed by pre-operative coronary angiography. Hypercholesterolemia was present in 4/7, diabetes mellitus in 3/7, hypertension in 3/7, obesity in 3/7, smoking in 2/7, and chronic renal failure in 0/7 patients. CPC were collected as the explant outgrowth from *ex vivo* cultured atrial specimens within 14 days. They were cultured in Iscove Modified Dulbecco's Medium supplemented with 20% FBS and 1% penicillin/streptomycin (all from GIBCO, Thermo Fisher Scientific, Waltham, MA, USA). Bone marrow mesenchymal stem cells (BM) and dermal fibroblasts (Fibro) were obtained from the same donors of CPC, and cultured as previously described 9. Primary neonatal cardiomyocytes were isolated from Wistar neonatal rats and used at post-natal days 1-3, as described 24. Human iPS cardiomyocytes were obtained from CPC, as described 25.

### siRNA transfection

CPC and cardiomyocytes were transfected with Lipofectamine RNAiMAX Reagent (Thermo Fisher Scientific). For periostin silencing, CPC (1×10^6^ cells) were transfected with siRNA against POSTN (30 pmol; Ambion, Thermo Fisher Scientific) in 800 µl of F12 medium (GIBCO) with 24 µl of lipofectamine. Next day, new medium was added. After 24 hrs, cells were washed and medium replaced with serum-free medium (DMEM High Glucose; GIBCO), and cells were cultured for 4 days. Naïve CPC-secreted Exo (ExoCPC) were processed using the same protocol, except for siRNA replacement by PBS. The same protocol was used for MirVana^TM^ miRNA mimic and cel-miR39 (both 30 pmol; Ambion) transfection. For YAP silencing, freshly isolated cardiomyocytes were transfected with siRNA against YAP (30 pmol; Ambion), as described above.

### Exo production and isolation

Exo were isolated from media conditioned by CPC, BM or Fibro cultured in serum-free DMEM High Glucose medium (GIBCO) for 4 days. Conditioned medium from 2×10^6^ cells was centrifuged at 3,000 g for 15 min, concentrated to 1 mL using Amicon Ultra-15 30-kDa filter (Merck Millipore, Burlington, MA, USA), filtered through a 0.22 µm membrane, and then centrifuged at 10,000 g for 15 min. The clean centrifugate (1 mL) was loaded on SEC columns (qEVoriginal from IZON Burnside, Christchurch, New Zealand) and fractions 8-9-10-11 (500 µl each) were collected. Purified Exo from 5 to 6 individuals were pooled for use *in vitro* and *in vivo*.

### Nanoparticles tracking analysis (NTA)

Exo were analyzed by NTA using NanoSight LM10 (Malvern Instruments, Malvern, UK), as per manufacturer's instructions.

### Immune-gold labeling and cryo-electron microscopy (cryo-EM)

Exo isolated by SEC were resuspended in 30 mM HEPES, pH 7.4, containing 100 mM KCl. For immune-gold labeling, Exo were incubated with an anti-periostin antibody (sc-398631; SantaCruz Biotechnology, CA, USA), and processed as described 26. Low-dose (<10 e-/Å2) imaging was done using a Talos 200C S/TEM (Thermo Fisher Scientific), equipped with a 4x4k Ceta camera. Digital micrographs were acquired at nominal magnifications of 36,000× and 45,000× (4.1 and 3.2 Å/pixel, respectively).

### Western blot analyses

Total proteins were extracted by lysing samples with ice-cold RIPA buffer supplemented with SIGMAFAST™ Protease Inhibitors and Phosphatase Inhibitor Cocktail 3 and 2 (all from Sigma, St. Louis, MI, USA). Nuclear proteins were extracted using a specific cell lysis buffer (1 mM PMSF; 0.1 M DTT, 50 mM; KCl 20 mM; Tris-HCl; pH 7.8) with 1:10 SIGMAFAST™ Protease Inhibitors (Sigma), as described 27. After vortexing, equal amounts of the same buffer and 1,2% NP-40 (Sigma) were added. After centrifugation, the lysate supernatant was collected as the cytosolic fraction, and pellet (nuclear fraction) was resuspended in buffer supplemented with NP-40 and sonicated 10 seconds for protein resuspension. The F-actin/G-Actin *In vivo* Assay Biochem Kit (Cytoskeleton, Denver, CO, USA) was used as per manufacturer's instructions. Total protein concentration were determined using BCA (Thermo Fisher Scientific). Proteins were boiled with Laemmli SDS sample buffer 6x (VWR International, Dietikon, Switzerland), separated on 4-20% Mini-PROTEAN®TGX™ Precast Gel, and transferred onto a PVDF membrane with a semi-dry transfer system (all from Bio-Rad Europe, Basel, Switzerland). Membrane were incubated with appropriate antibodies (Table S3), and then with IRDye® 680RD or 800CW goat anti-mouse or goat anti-rabbit secondary antibody (LI-COR Biosciences, Lincoln, Nebraska, USA). Total protein staining was performed using Revert™ 700 Total Protein (LI-COR Biosciences). Infrared signal was detected using Odyssey CLx Detection System (LI-COR Biosciences).

### ELISA

Periostin levels in intact or lysate Exo (1×10^9^ particles) were measured using human POSTN ELISA kit (Thermo Fisher Scientific), as per manufacturer's instructions.

### Flow cytometry

Surface markers expression on CPC and Exo was analyzed by flow cytometry. For cell analysis, untreated CPC or SiPOSTN-transfected CPC were harvested at day 5 (the time point of Exo collection) and labeled with CD73, CD105, CD90, CD34, CD16, CD45, and HLA-DR antibodies (all from Biolegend, San Diego, CA, USA). Exo markers were analyzed as previously described 28. EdU incorporation by rat cardiomyocytes was analyzed with Click-iT™ EdU assay for flow cytometry (Thermo Fisher Scientific), as per manufacturer's instructions. Samples were acquired with CytoFLEX (Beckman Coulter) and analysis was performed with Kaluza (Beckman Coulter).

### Cell proliferation assay

Proliferation rates of naïve CPC and SiPOSTN-transfected CPC were analyzed using Cell Counting Kit-8 (Dojindo, Kumamoto, Japan), as per manufacturer's instructions.

### MACSPlex analysis

Exo from naïve CPC and Exo from SiPOSTN-transfected CPC (ExoCPC_SiPOSTN) underwent immunocapturing with microbeads, before flow cytometry analysis using MACSPlex human Exosome Kit (Miltenyi; Bergisch Gladbach, Germany). Briefly, Exo were incubated with 37 fluorescently labeled capture bead populations, each coated with a specific antibody binding the respective surface epitope, and 2 control bead populations, followed by Exo detection reagent consisting of fluorescently labeled antibodies for CD9/CD63/CD81. Median fluorescence intensity (MFI) was measured on a MACSQuant‐Analyzer‐10 flow cytometer (Miltenyi), as described 29. All markers were analyzed simultaneously. The protocol was validated previously 30.

### Proteomic analysis

Proteomic data were obtained as previously described 9. The identified proteins were ranked based on their abundance estimated by normalized peptide spectrum match (nPSM) 31, whereby PSM was normalized for protein length by applying the formula nPSM=PSM/MW(KDa)*100.

### RNAse-A treatment

For RNAse-A treatment, Exo (5*10^9^) plus 0,1% of Triton-X (Sigma) was treated with 0,5 µg/µl of RNAse-A (Thermo Fisher Scientific) for 30 min at 37 °C 32. For functional experiments, RNAse-A- treated Exo were purified by SEC columns for removing Triton-X and RNAse-A excess.

### *In vitro* experiments

Neonatal rat cardiomyocytes were plated onto a 96-well plate and treated with 25×10^6^ Exo particles/well (62.5×10^6^ particles/cm^2^), corresponding to ~1 ng of periostin/well (as measured by ELISA), under serum-free condition. An equivalent particle number of ExoCPC_siPOSTN was used for comparison. Incubation times are shown in Figure S8. Cell proliferation was assessed by Click-iT™ EdU Cell Proliferation Kit for Imaging (Thermo Fisher Scientific) and by immunostaining for pH3, Ki67, Aurora B-kinase using the indicated antibodies (Table S3). Recombinant human full-length periostin (POSTN-FL) and short-length periostin peptide (POSTN_22-669_) were purchased from R&D (MN, USA; Cat: 3548-F2) and BioVendor (Brno, Czech Republic; Cat: RD172045025), respectively. FAK phosphorylation inhibitor, PF-573228 (10 mM; Sigma), was added to cells 30 min before Exo treatment.

### Exo fluorescent labeling

CPC conditioned medium (1 mL) was labeled with 5 µl of lipophilic commercial dye DiR (5 mg/mL; Thermo Fisher Scientific) and incubated at 37 °C for 5 min under gently shaking. Exo were then isolated by SEC. As a control, PBS (1 mL) with DiR (5 mg/mL) was loaded into SEC column and fractions corresponding to Exo containing fractions were collected and used as a control in cardiac retention experiments.

### *In vivo* experiments

Neonatal Wistar rats were injected IP with a cumulative dose of 6×10^9^ Exo particles, corresponding to ~240 ng periostin. Daily injections were performed from postnatal day 0 to day 3. An equivalent particle number of ExoCPC_siPOSTN was used for comparison. EdU (5-ethyl-2′-deoxyuridine; 350 ng cumulative dose) was injected at postnatal days 1 and 2 (Figure 5E/D). MI was induced in male Wistar rats (250-300g Body weight) anesthetized with a cocktail of Ketamine (Ketasol 100, 100 mg/kg) and Xylazine (Rompun 2%, 80 mg/kg), intubated, and ventilated. The left coronary artery was permanently ligated near its origin. After coronary occlusion, the peri-infarct myocardial region was injected at three different sites with PBS (0.1 mL total vol.) containing 10^11^ Exo particles of each type (corresponding to ~4 µg of periostin in the ExoCPC group), or PBS alone (control). EdU (3 µg cumulative dose) was injected IP at days 0, 2, 4, and 6 post-infarction (Figure 6A). Transthoracic echocardiography was performed at days 1 and 14 using Vevo2100 system (VisualSonic System 2100, FUJIFILM VisualSonics, Toronto, Canada) equipped with a 15-MHz linear transducer. LV ejection fraction (LVEF) was measured by Simpson's analysis, as previously described 26.

### Immunofluorescence and histology

Cryosections were stained with primary antibody overnight (for antibody list, see Table S3). Alexa Fluor secondary antibody (Thermo Fisher Scientific) was used for detection. Wheat germ agglutinin (WGA) antibody (Thermo Fisher Scientific) was used for cardiomyocyte cross-section area analysis, as per manufacturer's instructions. Images were acquired with Lionheart FX (Biotek, Winooski, VT, USA).

### Immunofluorescence analysis

Immunostained cell culture images were acquired with Lionheart FX automatic microscopy at 10× magnification and analyzed with Gen5 software (Biotek). A first mask on cTnI^+^ or α-actinin^+^ cells was created for identification of cardiomyocytes. For EdU and pH3 analyses, a secondary nuclear mask was created for nuclear signal detection in cTnI^+^ or α-actinin^+^ cells. For Ki67 and YAP analyses, nuclear MFI values of cTnI^+^ or α-actinin^+^ cells were measured.

### RNA extraction, reverse transcription and real-time PCR

Rat neonatal cardiomyocytes and dispersed cardiomyocytes obtained by perfusing infarcted hearts in a Langendorff-mode were lysed with TRI Reagent (Sigma), as per manufacturer's instructions. The pellet was air-dried, re-suspended in DEPC water, and RNA was quantified with NanoDrop™ 2000c (Thermo Fisher Scientific). RNA (500 ng) was reverse-transcribed using GoScript™ Reverse Transcription System (Promega Madison, Dübendorf, Switzerland). Real-time analysis was performed on CFX connect Bio-Rad Real-time PCR detection system (Bio-Rad). Data are shown as 2-∆∆Ct values. Couple of primers were as follows: Rat *Ccnd1* forward: TCAAGTGTGACCCGGACTG Reverse: GACCAGCTTCTTCCTCCACTT; Rat *Cdk1* forward: AACAGAGAGGGTCCGTTGTAA; Reverse: CACACCATAAGTCCCTTCTCC; Rat *Cdk4* forward: ACCGATCCCCGGTGTATG; Reverse: GGTTCATATCGAGTGGTAGCC; Rat *AurBk* forward: CATCGGACTAGGTTTCGGCT; Reverse: CATTCTCCAGGGCAAAAGCG; Rat *Gapdh* forward: TGCACCACCAACTGCTTAGC Reverse: GGCATGGACTGTGGTCATGAG. For analysis of periostin splice variants, cDNA was amplified from both CPC and POSTN-FL plasmid (BioCat, Heidelberg, Germany) using DreamTaq polymerase (Thermo Fisher Scientific,) using the following primers: *Postn* forward: GAAATCCCCGTGACTGTCTATA Reverse: TGGATTTTCACTGAGAACG. For miRNA quantification in Exo, as well as for cel-mir39 quantification in heart tissue, extracted RNA was reverse-transcribed with specific kits for miR16, miR132-3p, miR146a-5p, miR210, and cel-miR39 (TaqMan MicroRNA Reverse Transcription Kit, Applied Biosystem, Foster City, CA, USA). Real-time analysis was performed on CFX connect Bio-Rad Real-time PCR detection system using TaqMan (Thermo Fisher Scientific) and specific primers (TaqMan MicroRNA Assay, Applied Biosystems). In the experiment shown in Figure S5B, 100 ng of spike cel-mir39 was used as a normalizer. For cel-miR39 quantification in heart tissue, a cycle threshold value of 40 cycles was defined as negative on the control condition. Data are shown as 2^-∆∆Ct^ values.

### Statistical analyses

Results are shown as mean ± SEM (standard error of mean) from >3 independent experiments. Statistical analyses of differences between 3 groups were performed by one-way ANOVA followed by post-hoc Tukey's multiple tests, and those of differences between 2 groups were performed using unpaired t-test with Prism Version 7 GraphPad Software. Statistical significance was defined as *p*<0.05.

## Results

### CPC-secreted Exo express periostin on vesicular surfaces

**Figure 1:**
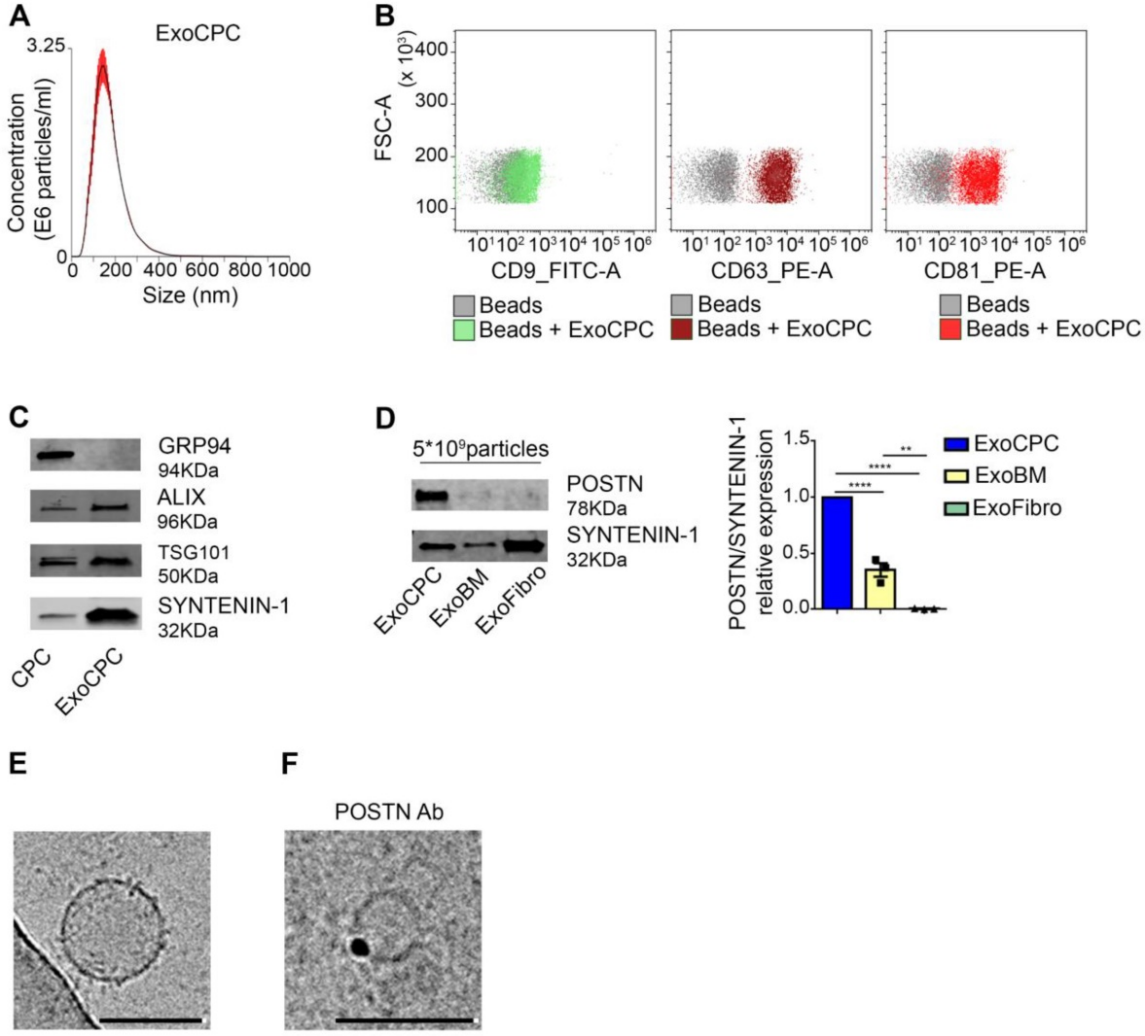
** CPC-derived Exo express exosome markers and periostin.** A. Nanoparticle tracking analysis of CPC-derived Exo (ExoCPC). B. Flow cytometry analysis of exosome surface markers (CD9, CD63, CD81) on ExoCPC (beads alone were used as a control). C. Western analysis of internal exosome markers (ALIX, tumor susceptibility gene 101 [TSG101], SYNTENIN-1) in ExoCPC and donor CPC. GRP94 was used as a purity control. D. Western analysis of periostin (POSTN) expression in ExoCPC, patient-matched bone marrow MSC-derived Exo (ExoBM), and dermal fibroblast-derived Exo (ExoFibro). Quantitative analysis of POSTN levels normalized for SYNTENIN-1 (*n* = 3; **** *p*<0.0001; ** *p*=0.001). E. Representative cryo-EM image of an ExoCPC particle. Scale bar: 200 nm. G. Cryo-EM image of an ExoCPC particle stained with anti-POSTN primary antibody and immune-gold showing POSTN detection on the vesicle surface. Scale bar: 200 nm.

Human CPC obtained as cardiac atrial appendage explant-derived cells using the *ex vivo* tissue culture technique displayed phenotypic and functional characteristics of mesenchymal/stromal progenitor cells, as described previously 8,9. Exo were isolated from serum-free medium conditioned by CPC using SEC 33 (Figure S1A). Soluble protein levels in the Exo fraction, as identified by presence of the exosomal protein SYNTENIN-1 33, were minimized by using a second round of SEC purification (Figure S1B). NTA of purified vesicles showed a mean diameter [range] of 117 ± 4.5 [∼40-400] nm (Figure 1A). Flow cytometry revealed expression of exosome surface markers (CD9, CD63, CD81; Figure 1B), and Western analysis revealed expression of internal exosome markers (SYNTENIN-1, ALIX, tumor susceptibility gene 101 [TSG101]) in purified CPC-secreted Exo (ExoCPC; Figure 1C). GRP94, used as a marker for intracellular material 33, was undetectable in the Exo fraction. These results indicated that CPC-secreted extracellular vesicles were enriched with exosomes. Having previously shown that CPC-secreted Exo express periostin 10, we then compared periostin expression levels in these Exo with those in Exo secreted by donor-matched BM cells or dermal fibroblasts using an anti-POSTN antibody (sc-398631; SantaCruz) raised against aa 537-836 mapping at the C-terminus of human periostin. ExoCPC expressed higher periostin levels compared to BM-secreted Exo, whereas dermal fibroblast-Exo did not express periostin at a detectable level (Figure 1D). Using cryo-EM, ExoCPC were visualized as round-shaped particles, ~50-200 nm in diameter (Figure 1E). Using anti-periostin as a primary antibody and immune-gold labeling, positive labeling appeared to be located on vesicle surfaces (Figure 1F). Periostin levels in ExoCPC measured by ELISA (Figure S2C) were comparable in intact Exo and Exo lysates (Figure S2D), consistent with selective presence of the protein on vesicle surfaces.

### CPC-secreted Exo selectively express a short periostin isoform

**Figure 2:**
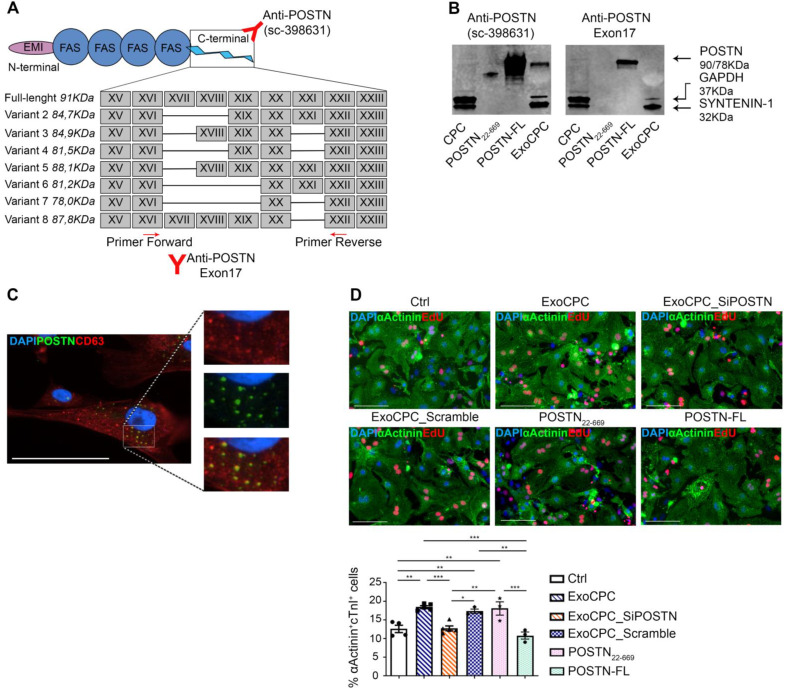
** CPC-derived Exo carry a short periostin isoform that promotes cardiomyocyte proliferation.** A. Schematic depicting the molecular structure of human periostin isoforms (EMI, EMI domain; FAS, fasciclin domain). Recognition sites for anti-POSTN Ab (sc-398631) and anti-POSTN-Exon17 Ab, as well as for the PCR primers used are indicated. B. Western analysis of periostin isoforms in donor CPC, a truncated periostin peptide (POSTN_22-669_), recombinant human full-length periostin (POSTN-FL), and ExoCPC. Periostin expression in ExoCPC was detected using anti-POSTN Ab (sc-398631), but not anti-POSTN-Exon17 Ab. C. Immunofluorescence photomicrograph showing co-localization of periostin and CD63 in CPC cells. Scale bar: 100 µm. D. Cultured neonatal rat cardiomyocytes immunostained for EdU (red) and cardiac-specific α-actinin (sarcomeric; green); nuclear staining with DAPI (blue). Scale bar: 100 µm. Cells were treated with naïve ExoCPC, Exo from CPC transfected with a siRNA against periostin (ExoCPC_SiPOSTN), Exo from CPC transfected with scramble siRNA (ExoCPC_Scramble), a truncated periostin peptide (POSTN_22-669_), recombinant human full-length periostin (POSTN-FL), or PBS (Ctrl). Quantitative analysis of EdU-positive cardiomyocytes (% of α-actinin-positive cells; *n* = 3 to 5 independent experiments; * *p*=0.0193; ** *p*=0.001,* p*=0.0206, *p*=0.0061,* p*=0.0024,* p*=0.0054; *** *p*=0.0007,* p*=0.0001 and* p*=0.0008).

Seven human periostin isoforms including the full-length protein (isoform 1; aa 1-811, *MW:* ~90 kDa) and isoforms lacking sequences between exons 17 and 21 have been identified (Figure 2A) 12,34. To assess the form of periostin carried by ExoCPC, we first performed a proteomic analysis, which identified human periostin isoform 7 precursor (corresponding to the deletion of exons 17, 18, 19 and 21) as the 6^th^ most highly enriched protein in these vesicles (Table S1). Consistent with this, Exo-carried periostin was not detected using an antibody raised against amino acids mapping at exon 17 (anti-POSTN-exon17) by SDS-PAGE (Figure 2B). The specificity of this antibody was demonstrated previously 35. This antibody elicited a positive signal in human full-length periostin peptide, as expected. Anti-POSTN (sc-398631) antibody showed a positive signal in ExoCPC, as mentioned above (Figure 1D/2B), as well as with recombinant human full-length periostin or a truncated periostin peptide (aa 22-669; POSTN_22-669_) 19. Of note, the periostin band in ExoCPC corresponded to a lower molecular weight (~78-kDa) than the full-length periostin band (~90-kDa), indicating a short periostin isoform (Figure S3B). Periostin levels in donor CPC were below Western blot detection threshold, suggesting that the protein may be swiftly incorporated into Exo for secretion. Immunofluorescence microscopy of CPC revealed periostin co-localization with CD63, consistent with exosomal secretion of the protein (Figure 2C). PCR analysis of CPC cDNA confirmed lack of full-length periostin transcripts (Figure S3A).

### CPC-secreted Exo induce cardiomyocyte proliferation *in vitro* via a short periostin isoform

**Figure 3:**
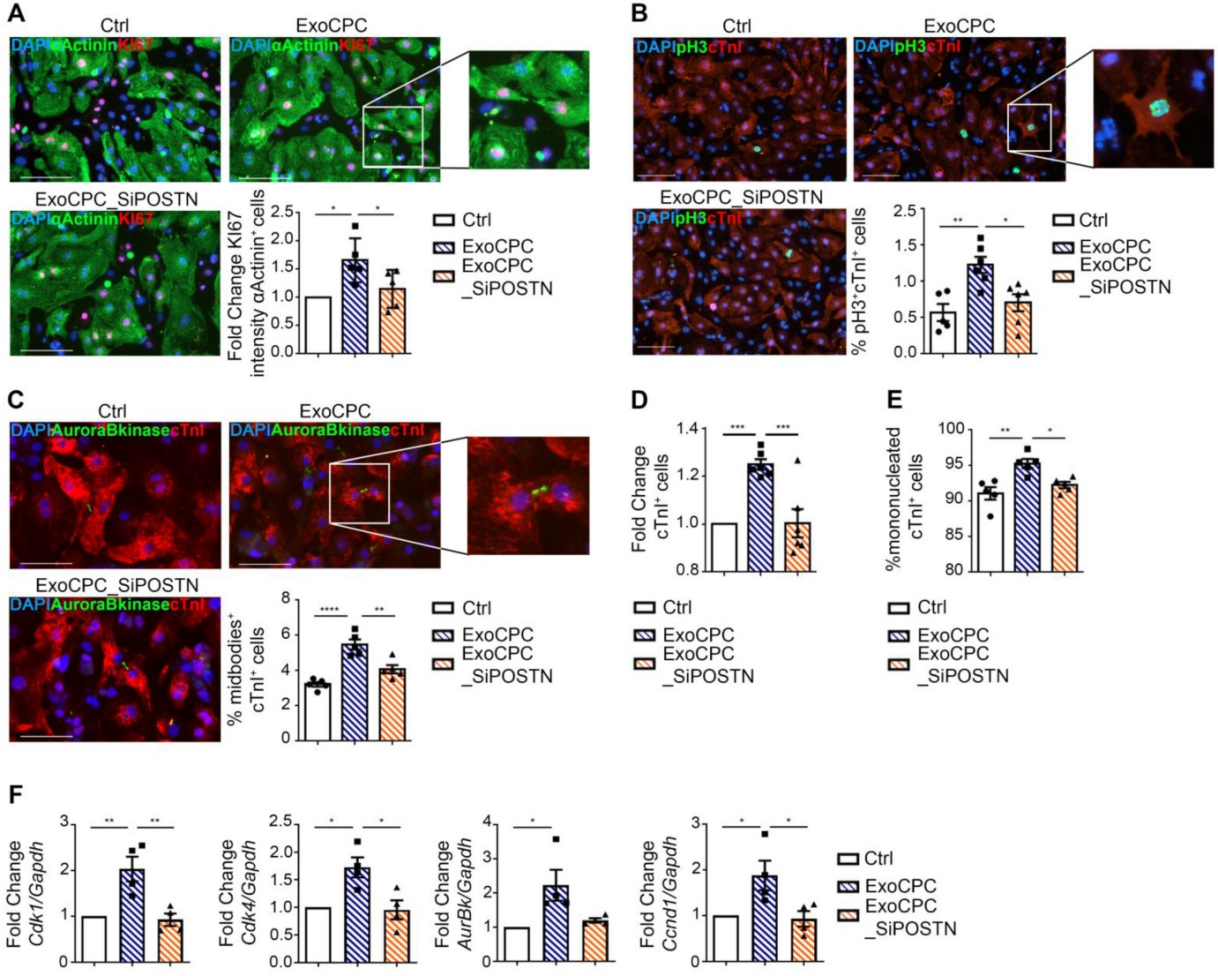
** CPC-derived Exo stimulate neonatal rat cardiomyocyte cycling through periostin.** A. Cultured neonatal rat cardiomyocytes immunostained for Ki67 (red) and cardiac-specific α-actinin (sarcomeric; green). Scale bar: 100 µm. Cells were treated with naïve CPC-derived Exo (ExoCPC), Exo from CPC transfected with a siRNA against periostin (ExoCPC_SiPOSTN), or PBS (Ctrl). Quantitative analysis of Ki67 nuclear fluorescence intensity in α-actinin-positive cells (fold-changes over Ctrl; *n* = 5; * *p=0.0115* and *p=0.0488*). B. Neonatal cardiomyocytes immunostained for phosphorylated histone H3 (pH3; green) and cardiac troponin I (cTnI; red). Scale bar: 100 µm. Quantitative analysis of pH3-positive nuclei (% of cTnI-positive cells; *n* = 5 to 6; * *p=0.0142;* ** *p*=0.0040). C. Neonatal cardiomyocytes immunostained for Aurora B-kinase (green) and cTnI (red). Scale bar: 100 µm. Quantitative analysis of Aurora B-kinase midbody expression (% of cTnI-positive cells; *n* = 5; ** *p*=0.0025; **** *p*<0.0001). D. Quantitative analysis of mononucleated cardiomyocytes (% of cTnI-positive cells; *n* = 5; *** *p*=0.0006 and *p*=0.0007). E. Quantitative analysis of cTnI-positive cells (fold-increase over Ctrl; *n* = 6; * *p*=0.0221; ** *p*=0.0026). F Real-time RT-PCR analysis of *Cdk1*; *Cdk4; AurBk* and *Ccnd1* mRNA expression in neonatal cardiomyocytes (fold-increase over Ctrl; *n* = 3; * *p*=0.0152,* p*=0.0108,* p*=0.0236,* p*=0.0391,* p*=0.0274; ** *p*=0.0058 and *p*=0.0038).

To investigate whether ExoCPC could induce proliferation of freshly isolated neonatal rat ventricular cardiomyocytes, and whether this effect could be periostin-dependent, CPC were transfected with a siRNA against periostin (SiPOSTN). CPC transfection with SiPOSTN resulted in the downregulation of the levels of its target protein in the secreted Exo by ~60% (Figure S2G). Transfection with siPOSTN did not affect CPC immunophenotype (Figure S2A) nor CPC proliferation rates (Figure S2B). It also did not affect levels of selected ExoCPC cargo molecules previously shown to participate in their cardioprotective effects, such as pregnancy-associated plasma protein-A (PAPP-A), miR132-3p, miR146a-5p, and miR210 9 (Figure S2F/I). Moreover, donor CPC transfection with siPOSTN did not alter ExoCPC surface marker expression (Figure S2H). Naïve ExoCPC and Exo from SiPOSTN-transfected CPC (ExoCPC_SiPOSTN) displayed comparable particle size profiles (Figure 1A; Figure S2E). Exo uptake by cardiomyocytes was assessed using Exo labeled with the DiR fluorescent dye. Cellular uptake efficiencies of naïve ExoCPC and ExoCPC_SiPOSTN were comparable (Figure S4). To assess DNA synthesis, cardiomyocytes were treated with EdU (10 µM), together with one of the following agents: naïve Exo (25*10^6^ particles, corresponding to ~1 ng of periostin), ExoCPC_SiPOSTN or Exo from CPC transfected with scramble siRNA (control; same dosage), recombinant human full-length periostin (~1 ng), or the truncated POSTN_22-669_ peptide (same dosage). Periostin expression in scramble siRNA Exo was confirmed by Western blotting (Figure S3C). A dose-response study showed dose-dependent ExoCPC effects on EdU incorporation in cardiomyocytes (Figure S5A). The lowest ExoCPC concentration that achieved peak EdU incorporation was used in subsequent *in vitro* studies. Naïve ExoCPC increased the number of EdU-positive nuclei in DNA-synthesizing cardiomyocytes, identified by cardiac-specific antigen expression (sarcomeric α-actinin or cardiac-specific troponin I; cTnI) and by their characteristic shape (Figure 2D). Similar results were observed using POSTN_22-669_ peptide, but not using ExoCPC_SiPOSTN or recombinant human full-length periostin (Figure 2D). Flow cytometry analysis of EdU incorporation (Figure S5E) confirmed the results of immunofluorescence microscopy. Naïve ExoCPC increased Ki67 fluorescence nuclear intensity by ~1.7-fold (Figure 3A), and the number of mitotic nuclei detected by an antibody specific for phosphorylated histone H3 (pH3) in condensed metaphase chromosomes 36 by ~2.2-fold (Figure 3B). Cytokinesis, the final step of the mitotic cell cycle, was analyzed by visualizing Aurora B-kinase, a component of the contractile ring at the site of cytoplasmic separation that is required for cytokinesis 37. Aurora B-kinase was detected after dissociation from the midbody, corresponding to the completion of cytokinesis. Naïve ExoCPC induced a ~1.7-fold increase in the number of cardiomyocytes staining positive for Aurora-B kinase at midbodies (Figure 3C). ExoCPC_SiPOSTN had no effects on Ki67 fluorescence nuclear intensity, pH3-positive nuclei, and Aurora B-kinase-positive cardiomyocytes. Naïve ExoCPC, but not ExoCPC_SiPOSTN, induced a ~1.25-fold increase in total cardiomyocytes (Figure 3D), and a ~1.05-fold increase in mononucleated cardiomyocytes (Figure 3E), with no changes in binucleated cardiomyocytes number (data not shown). These results were consistent with stimulation of cytokinesis by naïve ExoCPC. Moreover, a gene expression analysis of cyclins and cyclin-dependent kinases (Cdk) involved in the regulation of the cell cycle revealed upregulation of *Cdk1*, *Cdk4*, *AurBk, and Ccnd1* in response to naïve ExoCPC, but not in response to ExoCPC_SiPOSTN (Figure 3F). To assess the role of the exosomal miRNA cargo in cell cycle activity, ExoCPC were treated with RNAse-A. This treatment did not prevent the pro-proliferative effect of ExoCPC, although it slightly decreased it, suggesting a potential contributory role for miRNA cargo in cell cycle activity (Figure S5B-D). Collectively, these results indicate that ExoCPC induce cell cycling in freshly isolated neonatal rat cardiomyocytes through a short periostin isoform.

### CPC-secreted Exo stimulate cycling of human induced pluripotent stem (hiPS) cell-derived cardiomyocytes

**Figure 4:**
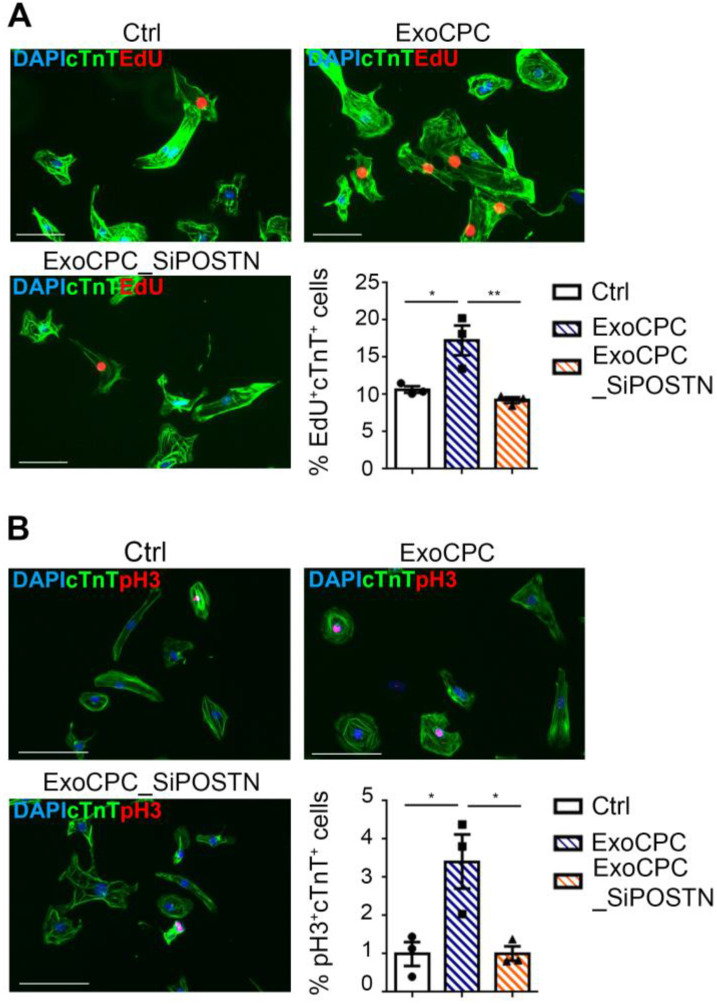
** CPC-derived Exo stimulate cycling of hiPS cell-derived cardiomyocytes.** A. hiPS cell-derived cardiomyocytes were treated with naïve CPC-derived Exo (ExoCPC), Exo from CPC transfected with a siRNA against periostin (ExoCPC_SiPOSTN), or PBS (Ctrl). Immunostaining for EdU (red) and cardiac troponin T (cTnT; green); nuclear staining with DAPI (blue). Scale bar: 100 µm. Quantitative analysis of EdU-positive nuclei of cTnT-positive cells (% of total cTnT-positive cells; *n* = 3; * *p*=0.0194; ** *p*=0.0079). B. hiPS cell-derived cardiomyocytes immunostained for phosphorylated Histone 3 (pH3; red) and cTnT (green). Scale bar: 100 µm. Quantitative analysis of pH3-positive nuclei of cTnT-positive cells (% of total cTnT-positive cells; *n* = 3; * *p*=0.0227 and *p*=0.0231).

hiPS cell-derived cardiomyocytes provided an *in vitro* assay platform to investigate the pro-proliferative activity of ExoCPC within a species-specific context. Naive ExoCPC, but not ExoCPC_SiPOSTN, induced ~1.6-fold and ~3.4-fold increases in EdU-positive and pH3-positive hiPS cell-derived cardiomyocytes, respectively (Figure 4A/B). Immunofluorescence microscopy results on EdU incorporation were confirmed by flow cytometry analysis (Figure S5F). These results indicate that ExoCPC stimulate cell cycle-reentry in iPS cell-derived cardiomyocytes of human origin.

### CPC-secreted Exo stimulate neonatal rat cardiomyocyte cycling *in vivo*

**Figure 5:**
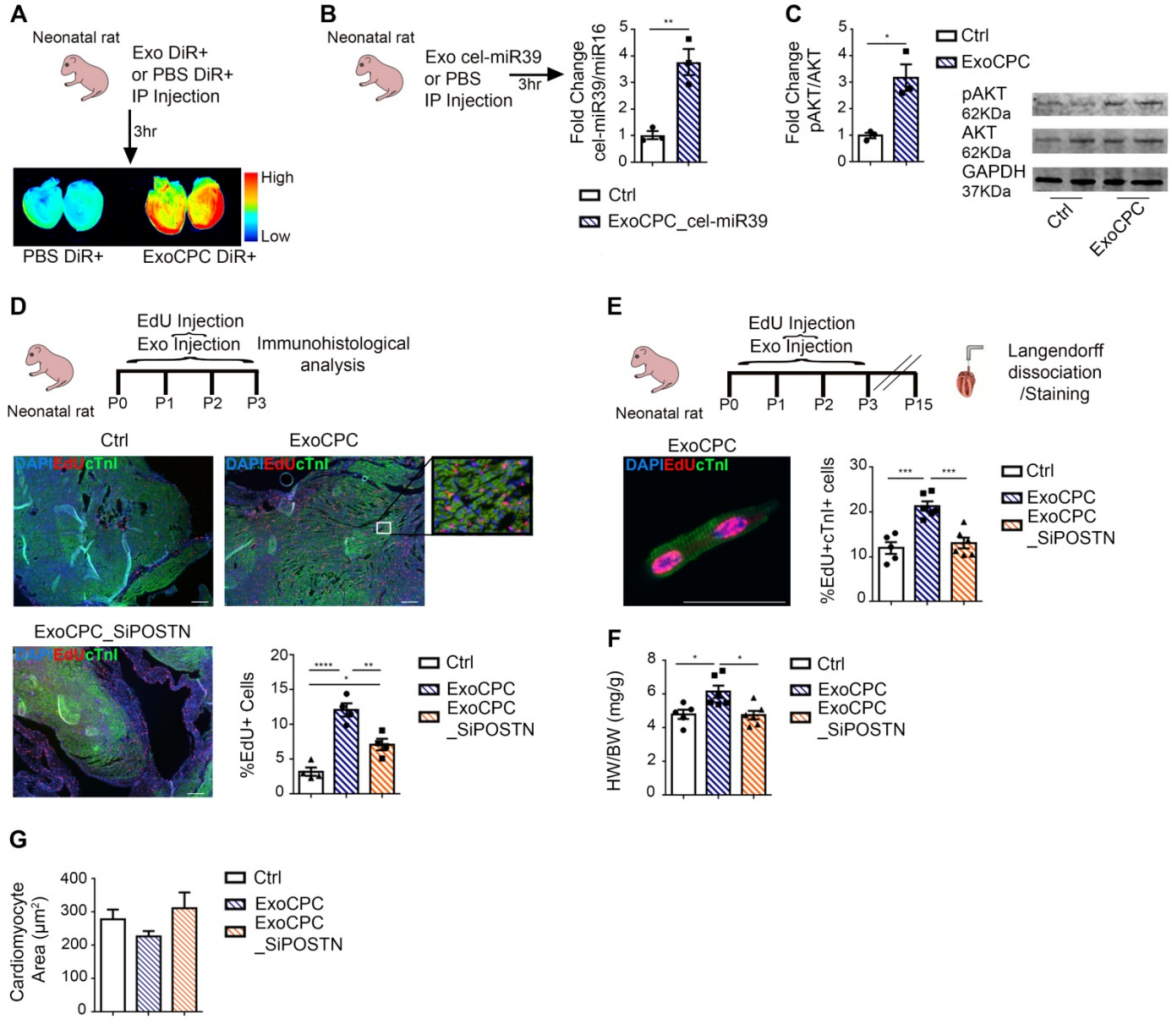
** CPC-derived Exo induce *in vivo* neonatal rat cardiomyocyte cycling via periostin.** A. IP administration of CPC-derived Exo (ExoCPC) labeled with DiR+ fluorescent dye (ExoCPC-DiR+), or DiR+ dye alone (PBS DiR+) in neonatal rats. Whole-heart laser scanner images of fluorescence intensity in rat hearts. B. Real-time RT-PCR analysis of cel-miR39 in heart tissue of rats injected IP with Exo from CPC transfected with cel-miR39 (ExoCPC_cel-miR39) or PBS (Ctrl). Quantitative analysis (fold-changes over Ctrl; *n* = 3; ** *p*=0.0060). C. Western analysis of phosphorylated Akt (pAkt) in heart tissue from rats injected IP with ExoCPC or PBS. Quantitative analysis (fold-changes in pAkt over Ctrl; *n* = 3; * *p*=0.0129). D. Heart sections from neonatal rats injected IP with naïve ExoCPC, Exo from CPC transfected with a siRNA against periostin (ExoCPC_SiPOSTN), or PBS, together with EdU, from day 0 to 3. Immunostaining for EdU (red) and cardiac troponin I (cTnI; green); nuclear staining with DAPI (blue). Scale bar: 200 µm. Quantitative analysis of EdU-positive nuclei (*n* = 4; * *p*=0.0189; ** *p*=0.0046; **** *p*<0.0001). E. Dispersed isolated neonatal cardiomyocyte (day 15 after ExoCPC injection) immunostained for EdU (red) and cTnI (green). Scale bar: 50 µm. Quantitative analysis of EdU-positive nuclei of cTnI-positive cells (% of total cTnI-positive cells) at day 15 after ExoCPC, ExoCPC_SiPOSTN, or PBS administration (*n* = 5 to 6; *** *p*=0.0002 and* p*=0.0005). F. Quantitative analysis of heart weight/body weight ratio (mg/g) at day 15 in neonatal rats that had been injected IP with ExoCPC, ExoCPC_SiPOSTN, or PBS, together with EdU, from day 0 to 3 (*n* = 5; * *p*=0.0225 and* p*=0.0135). G. Quantitative analysis of cardiomyocyte cross-section area (µm^2^) at day 15 after ExoCPC, ExoCPC_SiPOSTN, or PBS administration.

Because neonatal rat cardiomyocytes physiologically exhibit cell cycling activity 38, we evaluated whether ExoCPC could enhance this activity *in vivo*. As cardiac retention of systemically injected Exo is generally poor 39, we first validated the IP Exo delivery protocol using DiR-labeled ExoCPC or cel-miR39-transfected ExoCPC in neonatal hearts. Cardiac retention of IP injected DiR-labelled ExoCPC or cel-miR39-ExoCPC was demonstrated by whole-heart laser scanner analysis (Figure 5A) and cel-miR39 real-time RT-PCR analysis (Figure 5B), respectively. To demonstrate that ExoCPC taken up by hearts were functionally active, we measured the phosphorylation of Akt, a known molecular target of ExoCPC 9, in hearts. IP injection of ExoCPC induced a ~3.2-fold increase in Akt phosphorylation in the myocardium (Figure 5C). Next, we injected rats at post-natal day 0 with daily IP doses of EdU and naïve ExoCPC, ExoCPC_SiPOSTN (tot 6×10^9^ particles; equivalent to ~240 ng of periostin) or PBS (control) from day 0 to 3. Naïve Exo induced a significantly higher increase in the number of EdU-positive cells at day 3, compared with ExoCPC_SiPOSTN (Figure 5D). In separate experiments, neonatal rats were sacrificed at day 15 for EdU incorporation analysis in dispersed isolated cardiomyocytes for unambiguous cell type identification. Naïve ExoCPC, but not ExoCPC_SiPOSTN, induced a significant increase in heart weight (Figure 5F) and a ~1.8-fold increase in EdU-positive nuclei in dispersed isolated cardiomyocytes (Figure 5E and Figure S7A). The heart weight/body weight ratio was increased in ExoCPC-treated hearts, whereas cardiomyocyte cross-section area was not (Figure 5G), indicating lack of cardiomyocyte hypertrophy. These results suggest that ExoCPC may enhance physiological *in vivo* cardiomyocyte hyperplasia in neonatal rats via periostin.

### CPC-secreted Exo stimulate adult cardiomyocyte cycling in the infarct border region *in vivo*

**Figure 6:**
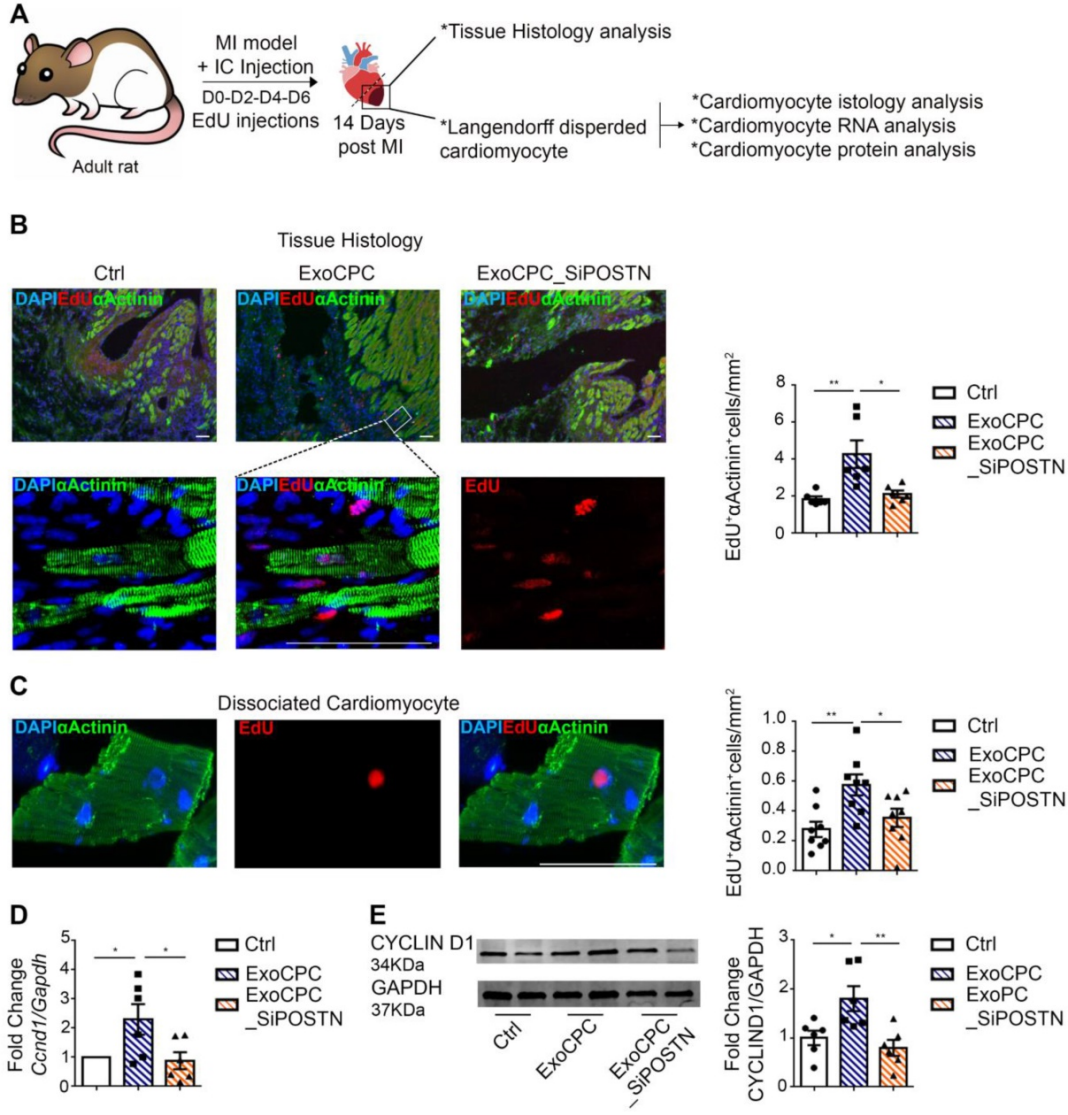
** CPC-derived Exo induce *in vivo* adult rat cardiomyocyte cycling in the infarct border region.** A. Schematic depicting of the experimental protocol. Myocardial infarction (MI) was induced by permanent ligation of the left coronary artery. Naïve CPC-derived Exo (ExoCPC), Exo from CPC transfected with a siRNA against periostin (ExoCPC_SiPOSTN), or PBS were injected in the myocardial region bordering the infarcted area. EdU was injected IP at days 0-2-4 and 6. Hearts were harvested at day 14. B. Heart sections immunostained for EdU (red) and cardiac-specific α-actinin (sarcomeric; green); nuclear staining with DAPI (blue). Scale bar: 50 µm. Quantitative analysis of EdU-positive nuclei of α-actinin-positive cells (% of total α-actinin-positive cells; *n* = 6; * *p*=0.0110; ** *p*=0.0047). C. Dispersed isolated cardiomyocytes from hearts at day 14 post-MI immunostained for EdU (red) and cardiac-specific α-actinin (green). Scale bar: 50 µm. Quantitative analysis of EdU-positive nuclei of α-actinin-positive cells (*n* = 8; * *p*=0.0482; ** *p*=0.0068). D. Real-time RT-PCR analysis of *Ccnd1* mRNA expression in dispersed isolated cardiomyocytes at day 14 post-MI (*n* = 6; * *p*=0.0461 and* p*=0.0288). E. Western analysis of cyclin D1 protein expression in dispersed isolated cardiomyocytes at 14 day post-MI. Quantitative analysis (fold-changes in cyclin D1 expression over Ctrl; *n* = 6; * *p*=0.0268; ** *p*=0.0063).

To investigate whether CPC-secreted Exo promote adult cardiomyocyte cycling *in vivo*, 10^11^ particles of either naive ExoCPC (equivalent to ~4 µg of periostin) or ExoCPC_SiPOSTN were injected into the infarct border region immediately after permanent ligation of the left coronary artery in adult rats (Figure 6A). EdU was delivered IP every two days (from days 0 to 6). Hearts were harvested at day 14, and EdU incorporation was analyzed both on heart sections and on dispersed isolated cardiomyocytes after α-actinin immunostaining. By histological analysis, naïve ExoCPC induced a ~2.3-fold increase in EdU-positive cardiomyocytes in the infarct border region, compared with control hearts, whereas ExoCPC_SiPOSTN were inert in this regard (Figure 6B). Similar results were observed in dispersed isolated cardiomyocytes from the same region (Figure 6C and Figure S7B). Naïve ExoCPC, but not ExoCPC_SiPOSTN, upregulated *Ccnd1* gene expression (Figure 6D) while also inducing an increase in cyclin D1 protein levels (Figure 6E) in dispersed isolated cardiomyocytes. There also were trends towards an upregulation of *Cdk1*, *Cdk4* and *AurBk* in hearts injected with naïve ExoCPC (Figure S6A). These results suggest that ExoCPC may stimulate cycling of adult cardiomyocytes in response to myocardial infarction via periostin-mediated upregulation of cyclins and Cdk. Compared with control hearts, infarct scar measured on Masson-trichrome-stained sections was significantly reduced following naïve ExoCPC treatment, but not following ExoCPC_SiPOSTN treatment (Figure S6C). Echocardiography studies were performed at days 1 and 14 post-MI. At day 1, left ventricular ejection fraction (LVEF) did not significantly differ among groups (data not shown). Compared to controls, LVEF was significantly increased at day 14 after naïve ExoCPC injection with, but not after ExoCPC_SiPOSTN injection (Figure S6B; Table S2). LVEF and scar size were not significantly different between the two Exo groups, however (Figure S6B/C). These results likely reflect periostin-independent, cardioprotective and anti-fibrotic effects of ExoCPC. We previously showed that ExoCPC were beneficial in rat MI models, in part due to the fact that they carried PAPP-A, a cardioprotective protein 9. Here, we show that naïve ExoCPC and ExoCPC_SiPOSTN express similar levels of PAPP-A (Figure S2F and Figure S6D). While naïve ExoCPC induced significant increases in cardiomyocyte cycling and LVEF, along with a decrease in cardiac fibrosis, the observation that ExoCPC_SiPOSTN also tended to improve LVEF but did not affect cell cycle activity supports cardioprotection as the main mechanism of benefit of ExoCPC.

### CPC-secreted Exo stimulate cardiomyocyte cycling via phosphorylation of focal adhesion kinase

**Figure 7:**
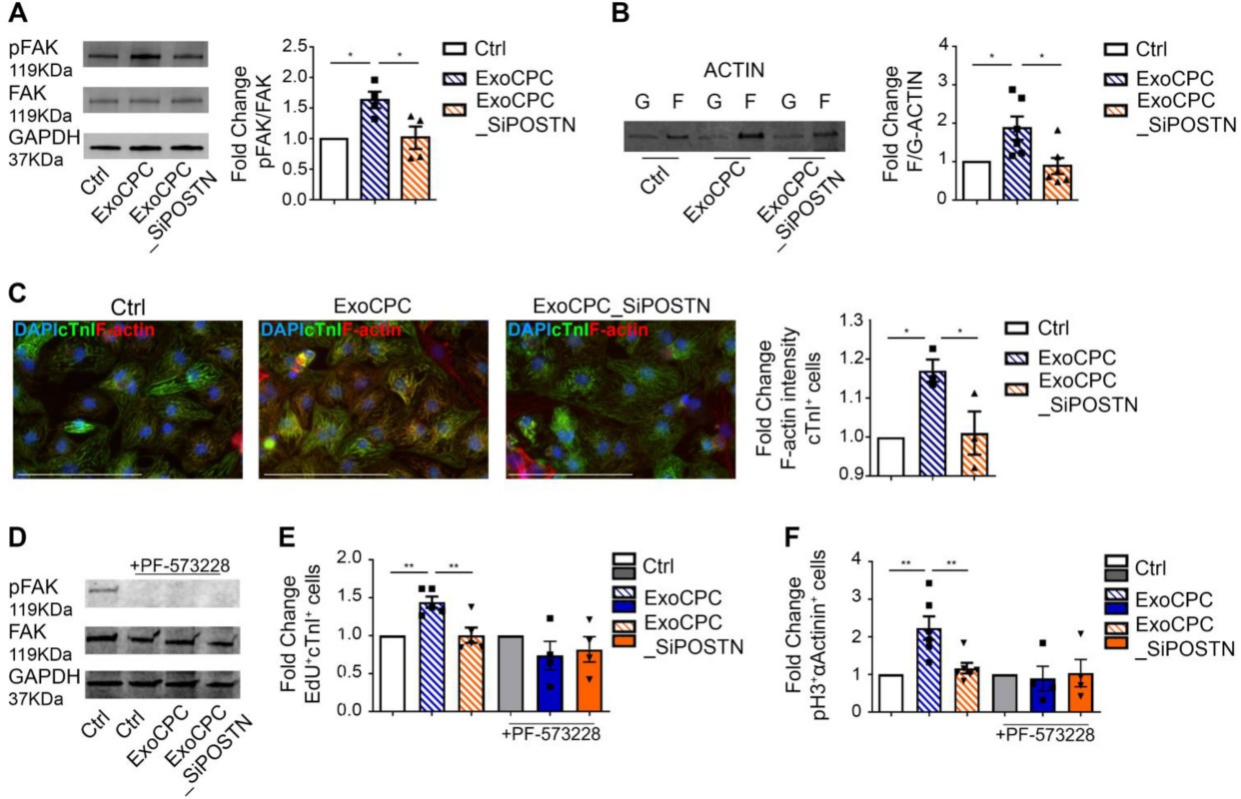
** CPC-derived Exo induce neonatal cardiomyocyte cycling via periostin-mediated FAK activation.** A. Western analysis of focal adhesion kinase (FAK) phosphorylation in neonatal cardiomyocytes treated with naïve CPC-derived Exo (ExoCPC), Exo from CPC transfected with a siRNA against periostin (ExoCPC_SiPOSTN), or PBS (Ctrl). Quantitative analysis of FAK phosphorylation (fold-changes over Ctrl; *n* = 4; * *p*=0.0187 and* p*=0.0220). B. Western analysis of F-actin and G-actin expression. Quantitative analysis of F-actin/G-actin ratio (fold-changes over Ctrl; *n* = 6; * *p*=0.0419 and* p*=0.0172). C. Quantitative analysis of F-actin intensity in cardiac troponin I (cTnI)-positive cells (fold-change over Ctrl; *n* = 3; * *p*=0.0356 and* p*=0.0455). D. Western analysis of the effect of the FAK phosphorylation inhibitor, PF-573228, on FAK phosphorylation. E. Quantitative analysis of the effect of PF-573228 on EdU-positive cardiomyocytes (fold-changes; *n* = 4 to 5; ** *p*=0.0064 and* p*=0.0048). F. Quantitative analysis of the effect of PF-573228 on phosphorylated histone H3 (pH3)-positive cardiomyocytes nuclei (fold changes; *n* = 4 to 6; ** *p*=0.0033 and* p*=0.0068).

Previous studies showed that periostin stimulates the phosphorylation of focal adhesion kinase (FAK), a non-receptor tyrosine kinase that regulates cell proliferation, survival and motility, through binding to integrins that function as periostin receptors in cardiomyocytes 18,19,40. Here, we showed that naïve ExoCPC, but not ExoCPC_SiPOSTN, stimulated the phosphorylation of FAK in neonatal rat cardiomyocytes (Figure 7A). This effect was associated with actin cytoskeleton rearrangement, as evidenced by an increased ratio between polymerized F-actin and its monomeric, G-actin form, by both Western blotting and immunochemistry (Figure 7B/C). To test whether phosphorylation of FAK was required for Exo-mediated cell cycle-reentry, cardiomyocytes were pre-treated with PF-573228, a specific inhibitor of FAK phosphorylation. PF-573228-mediated inhibition of FAK phosphorylation was confirmed by Western blotting (Figure 7D). PF-573228 treatment prevented ExoCPC-mediated nuclear EdU incorporation (Figure 7E) and pH3-positive cardiomyocytes increase (Figure 7F). These results indicate that ExoCPC stimulate cardiomyocyte cycling through phosphorylation of FAK.

### CPC-secreted Exo stimulate cardiomyocyte cycling via regulation of the Hippo pathway

**Figure 8:**
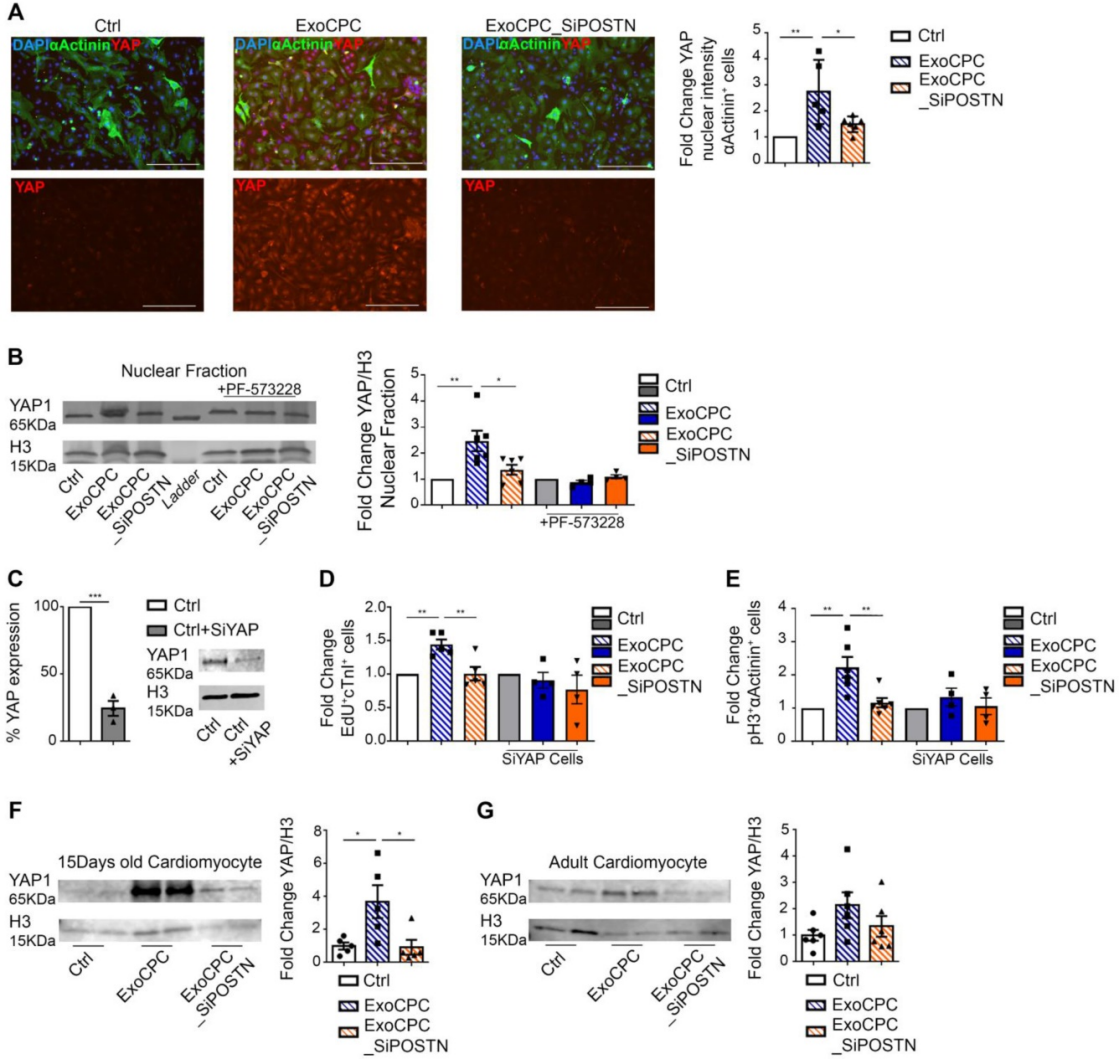
** CPC-derived Exo induce *in vitro* and *in vivo* cardiomyocyte cycling via periostin-mediated YAP nuclear translocation.** A. Cultured neonatal rat cardiomyocytes immunostained for YAP (red) and cardiac-specific α-actinin (sarcomeric; green); nuclear staining with DAPI (blue). Scale bars: 200 µm. Quantitative analysis of YAP fluorescence intensity in the nuclear fraction of cardiomyocytes treated with naïve CPC-derived Exo (ExoCPC), Exo from CPC transfected with a siRNA against periostin (ExoCPC_SiPOSTN), or PBS (Ctrl; *n* = 5; * *p*=0.0316; ** *p*=0.0049). B. Western analysis of YAP and histone H3 in the nuclear fraction of cardiomyocytes and PF-573228 pre-treated cardiomyocytes. Quantitative analysis of YAP levels normalized for H3 in the nuclear fraction (fold-changes over Ctrl; *n* = 4 to 6; * *p*=0.0240; ** *p*=0.0052). C. Knocking down of YAP expression in the nuclear fraction using a siRNA against YAP (SiYAP; fold-changes over naive cells; *n* = 3; *** *p*=0.0002). D. Quantitative analysis of the effect of SiYAP on EdU-positive cardiomyocytes (fold-changes over the respective controls; *n* = 4 to 5; ** *p*=0.0064 and* p*=0.0048). E. Quantitative analysis of the effect of SiYAP on phosphorylated H3 (pH3)-positive cardiomyocytes (fold-changes over the respective controls; *n* = 4 to 6; ** *p*=0.0033 and* p*=0.0068). F. Western analysis of YAP and H3 levels in dispersed isolated cardiomyocytes at day 14 after IP injection of ExoCPC, ExoCPC_SiPOSTN or PBS. Quantitative analysis of YAP levels normalized for H3 (fold-changes over Ctrl; *n* = 5; * *p*=0.0300 and* p*=0.0258). G. Western analysis of YAP and H3 levels in dispersed isolated cardiomyocytes from adult rat hearts explanted 14 days after MI and intramyocardial injection of ExoCPC, ExoCPC_SiPOSTN, or PBS. Quantitative analysis of YAP levels normalized for H3 (fold- changes over Ctrl; *n* = 6).

Phosphorylation of FAK and inhibition of filamentous actin depolymerization have been associated with the nuclear translocation of the Hippo pathway effector, the Yes-associated protein (YAP) transcriptional coactivator, in cardiomyocytes 41. We therefore investigated the effect of ExoCPC on the regulation of the Hippo signalling pathway. Naïve ExoCPC, but not ExoCPC_SiPOSTN, induced a ~2.7-fold increase in the YAP nuclear signal intensity in cardiomyocytes, as measured by immunofluorescence staining (Figure 8A), and a ~2.5-fold increase in YAP nuclear levels, as quantified by Western blotting (Figure 8B). PF-573228 abrogated Exo-mediated YAP nuclear translocation (Figure 8B) reflecting a requirement for FAK phosphorylation for this effect. A siRNA against YAP downregulated the levels of its target protein by ~75% (Figure 8C) and prevented ExoCPC-mediated increases in both EdU-positive and pH3-positive cardiomyocytes (Figure 8D/E). *In vivo*, IP administration of naïve ExoCPC, but not ExoCPC_SiPOSTN, at post-natal day 0 induced a ~3.7-fold increase in YAP levels in dispersed isolated cardiomyocytes from hearts harvested 2 weeks later (Figure 8F). There was a similar trend toward increased YAP levels in dispersed isolated cardiomyocytes from adult hearts injected with naïve ExoCPC at the time of MI and harvested 2 weeks later (Figure 8G). These findings suggest that ExoCPC may stimulate cardiomyocyte cycling via periostin-mediated regulation of the Hippo signaling pathway both *in vitro* and *in vivo*.

## Discussion

We previously showed that human CPC-secreted exosomes were enriched with periostin and improved cardiac function after MI. Here we show that these vesicles have cardiomyocyte cell cycle activity that is dependent on a short periostin isoform present on their surfaces. Indeed, CPC-secreted exosomes induced proliferation of cultured neonatal rat cardiomyocytes, and of human iPS cell-derived cardiomyocytes, as evidenced by increases in EdU incorporation, Ki67 nuclear levels, pH3-positive nuclei, and dissociation of Aurora B-kinase from the midbody (corresponding to the completion of cytokinesis in cardiomyocytes). *In vivo* delivery of CPC-derived exosomes enhanced physiological rat cardiomyocyte hyperplasia in neonatal rats, as well as cardiomyocyte cell cycle-reentry in peri-infarct myocardium in adult rats. The increase in mitotic markers was associated with upregulation of cyclines and cycline-dependent kinases (*Cdk1*, *Cdk4*, *AurBk, Ccnd1*) which are under the control of YAP transcription factor, along with overexpression of cyclin D1 protein in cardiomyocytes in the infarct border region. Knocking down of periostin in donor CPC nullified cell cycle activity of their secreted vesicles.

Mechanistically, periostin present on the surface of CPC-secreted exosomes stimulated the phosphorylation of FAK via integrin binding 19 to induce inhibition of filamentous actin depolymerization, activation of nuclear localization of YAP, and cardiomyocyte cell cycle-reentry. PF-573228, a chemical inhibitor of FAK phosphorylation, blocked YAP nuclear translocation and cardiomyocyte cycling in response to the exosomes. A siRNA against YAP elicited similar effects both *in vitro* and *in vivo*. It has been shown that pro-proliferative microRNAs (e.g., miR199a-3p) similarly stimulate nuclear localization of YAP in cardiomyocytes 41, and that inhibition of filamentous actin depolymerization by itself activates YAP nuclear translocation and induces adult cardiomyocytes to adopt a more proliferative state with fetal-like chromatin and transcriptional profiles 42.

CPC-derived exosomes were more highly enriched with periostin compared with BM-derived exosomes, whereas dermal fibroblast-secreted exosomes lacked this protein, reflecting donor cell type-specific differences. These results may reflect, in part, the fact that CPC were isolated as the cellular outgrowth of *ex vivo* cultured atrial explants. Because specimen procurement is associated with tissue injury, which triggers periostin expression in the adult heart 13-16, tissue procurement by itself may induce periostin expression. Moreover, because periostin regulates ECM remodeling and cell migration 12,22, and because the cellular outgrowth is comprised of cells that migrate out of tissue explants, it is perhaps not surprising that periostin-expressing cells are enriched in the cellular outgrowth.

Alternative splicing of the human periostin gene results in 6 isoforms lacking sequences between exons 17 and 21, in addition to full-length periostin 12,34. Periostin carried by CPC-secreted Exo was detected using an antibody (sc-398631) raised against aa 537-836 mapping at the C-terminus of human periostin, but not using an antibody raised against amino acids mapping at exon 17 35, indicating an exon 17-lacking isoform. The size of this protein was 78-kDa, whereas that of full-length periostin is 90-kDa. Moreover, full-length periostin transcripts were undetectable in CPC. In addition, proteomic analysis identified periostin isoform 7 precursor (corresponding to absence of exons 17, 18, 19 and 21) as the 6^th^ most abundant protein in CPC-exosomes. To further address the role of different forms of periostin in cardiomyocyte proliferation, we directly compared a truncated form of human periostin (aa 22-669), which was previously shown to induce cardiomyocyte proliferation 19, and recombinant human full-length periostin in neonatal rat cardiomyocytes. The truncated form of periostin induced cardiomyocyte cell cycle-reentry, whereas full-length periostin did not. These results are in line with a previous previous study in rats showing that gene expression levels of all 4 isoforms of rat periostin (i.e., Pn-1 as a full-length form, Pn-2 lacking exon 17, Pn-3 lacking exon 21, and Pn-4 lacking exons 17 and 21) reached a peak at 5 to 7 days post-MI. Selective inhibition of Pn-1 by a neutralizing antibody against periostin exon 17, which inhibited Pn-1 but not Pn-2/4, reduced infarct size and scarring while also preventing ventricular dilation, but did not affect cardiomyocyte proliferation 35. These findings indicated detrimental effects of full-length periostin on infarct size and cardiac remodeling, but not on cardiomyocyte proliferation. In line with these findings, here we show that short forms of periostin, but not full-length periostin, selectively stimulate cardiomyocyte cell cycle-reentry. These results help settle the long-lasting dispute as to whether periostin promotes cardiomyocyte proliferation. The effect may come down to specific isoform expressed.

Naïve CPC-exosomes significantly improved left ventricular ejection fraction while also reducing scar size after MI. Exosomes released from periostin-knockdown CPC exerted a similar effect on left ventricular function, which, however, did not reach statistical significance. We interpret these results as evidence for periostin-independent cardioprotective activities of CPC-exosomes. While significant, the magnitude of cardiomyocyte proliferation induced by naïve CPC-exosomes was limited. Accordingly, cardioprotection appeared to be the main mechanism of benefit, with proliferation also playing a contributory role. Nevertheless, these findings suggest a potential for exosomal short-length periostin in ischemic heart disease; however, further studies are needed to optimize the therapeutic benefit.

## Supplementary Material

Supplementary figures and tables.
