# Supplementary material for: An exosomal-carried short periostin isoform induces cardiomyocyte proliferation
Source: Theranostics. 2021 Mar 23;11(12):5634–49. doi: 10.7150/thno.57243 (PMC8058720; doi:10.7150/thno.57243)
Supplement: Supplementary file 1 — Supplementary figures and tables. [file thnov11p5634s1.pdf]

An exosomal-carried short periostin isoform induces cardiomyocyte proliferation

Carolina Balbi[1,2]*, Giuseppina Milano[1,3], Tudor E. Fertig[4], Edoardo Lazzarini[5], Sara Bolis[1,5], Yoshiaki Taniyama[6], Fumihiro Sanada[6], Dario Di Silvestre[7], Pierluigi Mauri[7], Mihaela Gherghiceanu[4], Thomas F. Lüscher[2,8], Lucio Barile[5,9,10], Giuseppe Vassalli[1,2,10]*.

[1]Laboratory of Cellular and Molecular Cardiology, Istituto Cardiocentro Ticino, Lugano, Switzerland;

[2]Center for Molecular Cardiology, University of Zurich, Zurich, Switzerland;

[3]Laboratory of Cardiovascular Research, Lausanne University Hospital, Lausanne, Switzerland;

[4]Victor Babes National Institute of Pathology, Bucharest, Romania;

[5]Laboratory for Cardiovascular Theranostics, Istituto Cardiocentro Ticino, Lugano, Switzerland;

[6]Department of Clinical Gene Therapy, Osaka University Graduate School of Medicine, Suita, Osaka, Japan;

[7]Proteomics and Metabolomic Lab, ITB-CNR, Segrate, Italy;

[8]Royal Brompton & Harefield Hospital, Imperial College, London, UK;

[9]Institute of Life Science, Scuola Superiore Sant'Anna, Pisa, Italy;

[10]Faculty of Biomedicine, Università della Svizzera Italiana (USI), Lugano, Switzerland.

***Address correspondence to:**

Prof. Giuseppe Vassalli, Laboratory of Cellular and Molecular Cardiology, Istituto Cardiocentro Ticino, Via Tesserete 48, 6900 Lugano. Tel: +41 091 8053359. E-mail: giuseppe.vassalli@cardiocentro.org

Dr. Carolina Balbi, Laboratory of Cellular and Molecular Cardiology, Istituto Cardiocentro Ticino, Via Tesserete 48, 6900 Lugano. Tel: +41 091 8053386. E-mail: carolina.balbi@cardiocentro.org

Supplementary Figures:

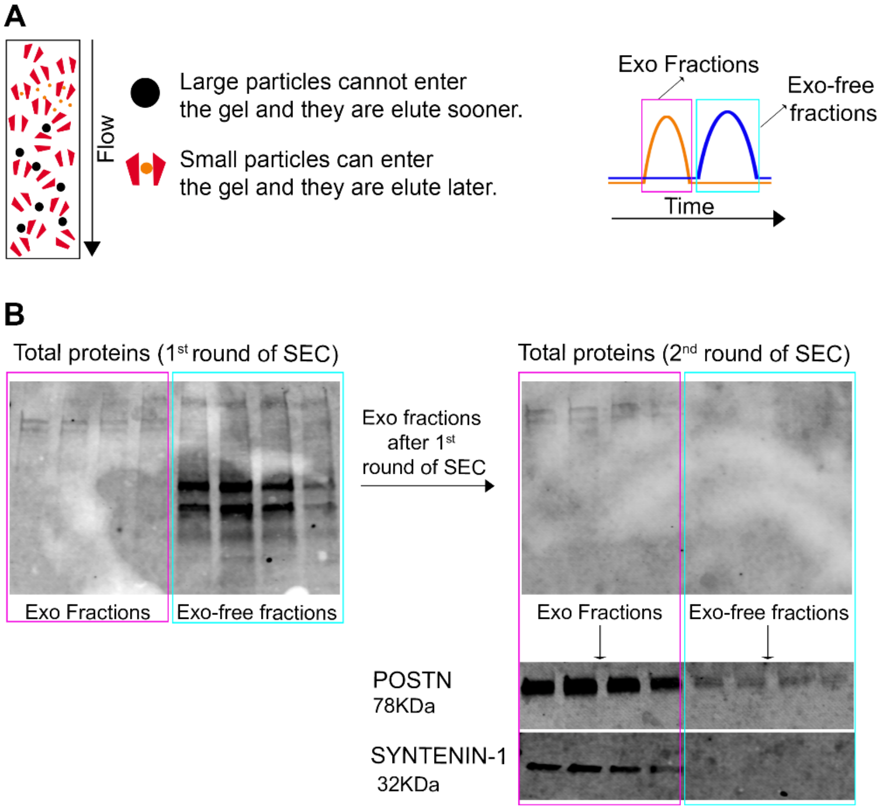

Figure S1: Periostin enrichment in exosome-containing SEC fractions of CPC conditioned medium

A Schematic of size exclusion chromatography (SEC) isolation principle. **B** Western blot analysis of total protein, periostin (POSTN) and exosome marker SYNTENIN-1. Left panel: Total protein staining after the first round of SEC purification. Right panel: Total protein, POSTN and SYNTENIN-1 after the second round of Exo SEC isolation. POSTN was enriched in Exo fractions, identified by high SYNTENIN-1 levels, vs. non-vesicular fractions.

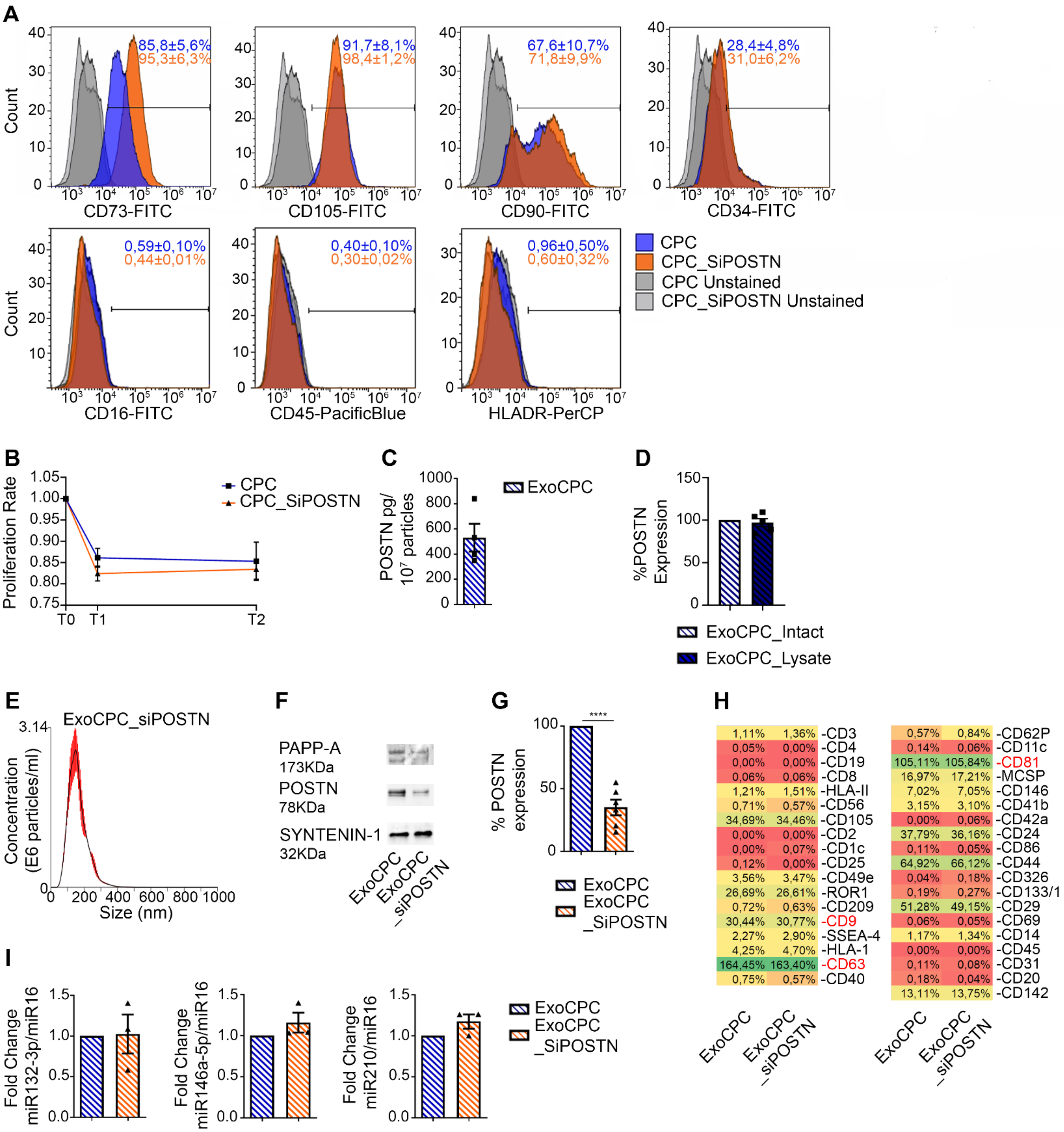

Figure S2: Characterization of periostin-silenced CPC and CPC-derived exosomes

A Flow cytometry analysis of surface antigen expression in naïve CPC and CPC transfected with a siRNA against periostin (CPC_SiPOSTN; *n* = 3). **B** Proliferation rates of naïve CPC and CPC_SiPOSTN at T0 (pre-transfection); T1 (24 hrs post-transfection) and T2 (5 days post-

transfection; n = 3). **C** POSTN quantification (pg/10^7 Exo particles) on intact ExoCPC by ELISA (n = 4). **D** POSTN levels in ExoCPC lysate vs. intact ExoCPC, measured by ELISA (% of levels in intact ExoCPC; n = 5). **E** Nanoparticle tracking analysis of Exo from CPC_SiPOSTN (ExoCPC_SiPOSTN). **F** Western analysis of pregnancy-associated plasma protein-A (PAPP-A)/pappalysin-1, POSTN, and SYNTENIN-1 in ExoCPC and ExoCPC_SiPOSTN. **G** POSTN levels, normalized for SYNTENIN-1 levels, in ExoCPC_SiPOSTN vs. naïve ExoCPC, as assessed by Western blotting (% of levels in naïve ExoCPC; n = 6; **** $p<0.0001$). **H** MACSPlex analysis of surface marker expression in ExoCPC and ExoCPC_SiPOSTN (green, yellow, and red colours indicate high, moderate, and low expression levels, respectively). **I** Real-time RT-PCR analysis of miR132-3p, miR146a-5p, and miR210 expression, in ExoCPC_SiPOSTN vs. ExoCPC (n = 3).

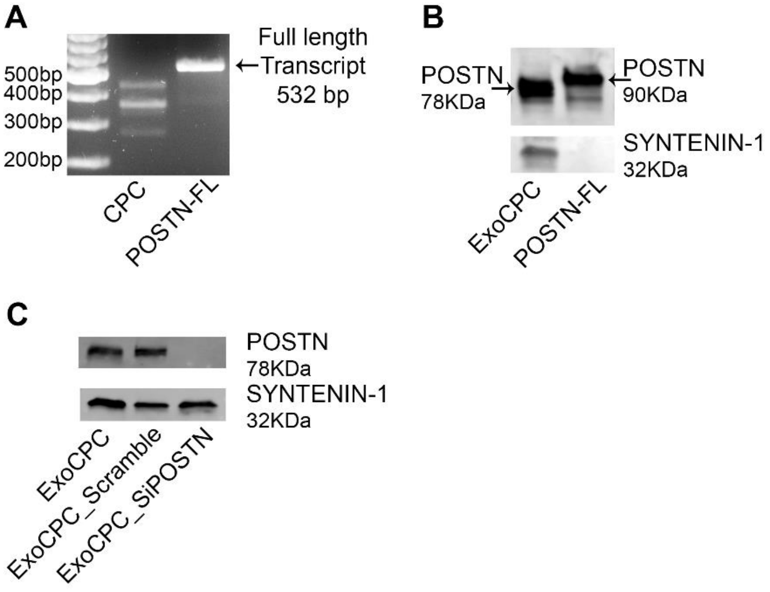

Figure S3: Assessment of POSTN transcripts in CPC and POSTN protein levels in exosomes from naïve, scramble-siRNA transfected, and periostin-silenced transfected CPC

A PCR analysis of human periostin (POSTN) on CPC cDNA. cDNA from a full-length human periostin (POSTN-FL) expression plasmid was used for comparison. **B** Western analysis of POSTN and SYNTENIN-1 (exosome marker) expression in ExoCPC. Recombinant human POSTN-FL was loaded for size comparison. **C** Western analysis of POSTN and SYNTENIN-1 expression in ExoCPC and in Exo from CPC transfected with scamble-siRNA (ExoCPC_Scramble), or with siRNA against periostin (ExoCPC_SiPOSTN).

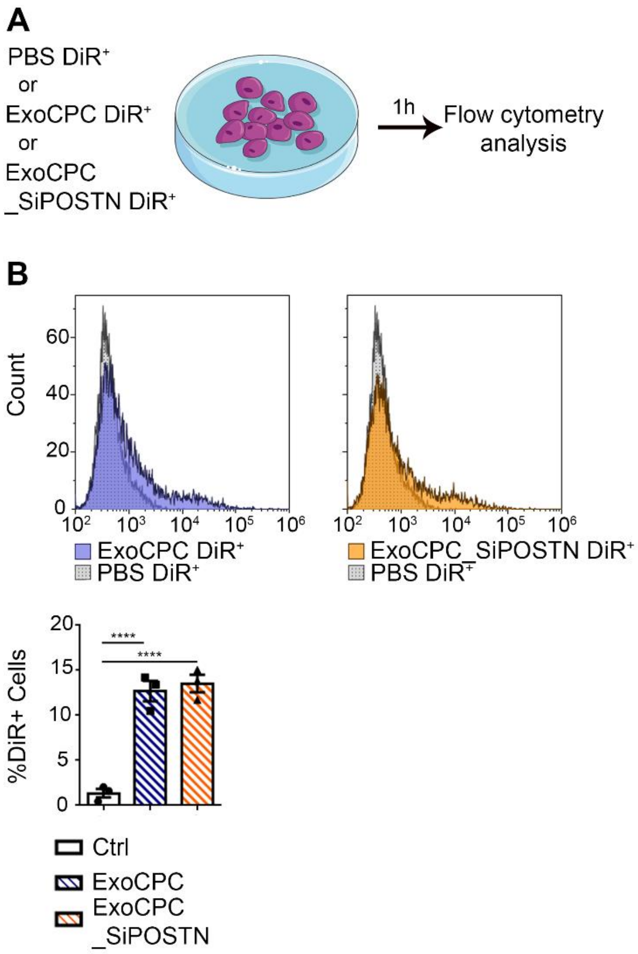

Figure S4: Periostin silencing does not affect exosome cellular uptake

A Schematic of the experimental protocol. CPC-derived Exo (ExoCPC) were labelled with DiR fluorescent dye (ExoCPC DiR$^+$) and re-purified by SEC to remove dye excess. DiR diluted in PBS at the same concentration used in ExoCPC, and processed with a SEC column, was used as a control (PBS DiR$^+$). ExoCPC DiR$^+$ or PBS DiR$^+$ were added to cultured neonatal rat cardiomyocytes, which were analysed by flow cytometry 1 hour later. **B** Quantification of DiR$^+$ cells after treatment with naïve ExoCPC or Exo from CPC transfected with a siRNA against periostin (ExoCPC_SiPOSTN; n = 3; **** $p<0.0001$).

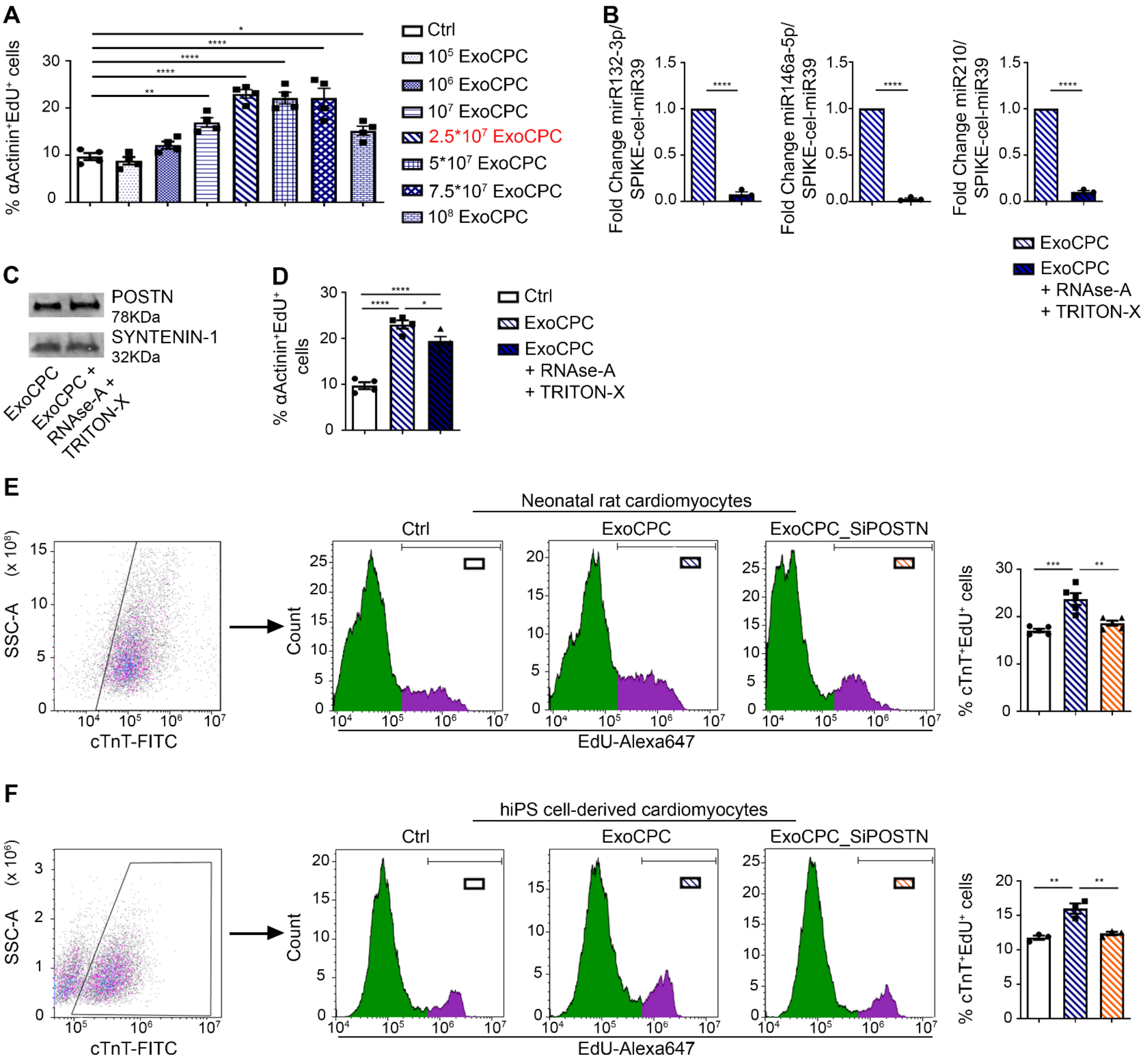

Figure S5: Exosome dose-response study, effect of miRNAs on cell cycle activity, and periostin-dependent effect of exosomes on rat neonatal cardiomyocyte and hiPS cell-derived cardiomyocyte proliferation

A Dose-response study of CPC-secreted Exo (ExoCPC) and neonatal rat cardiomyocyte cell cycle activity. Quantitative analysis of EdU-positive cardiomyocytes (% of cardiac α-actinin–positive cells; n = 4; * *p=0.0413; ** p=0.0033; **** p<0.0001*). A concentration of 2,5*10^7 particles/well was found to be most effective. **B** Real-time RT-PCR analysis of miR132-3p, miR146a-5p, and miR210 levels

in both untreated and RNAse-A–treated ExoCPC (fold-changes vs. ExoCPC; *n* = 3; **** *p<0.0001*). **C** Western analysis of POSTN and SYNTENIN-1 levels in both untreated and RNAse-A–treated ExoCPC. **D** Quantitative analysis of EdU-positive rat cardiomyocytes (% of αActinin-positive cells; *n* = 4; * *p=0.0417; **** p<0.0001*) treated with naïve CPC-derived Exo (ExoCPC), RNAse-A treated ExoCPC or PBS (Ctrl). **E** Flow cytometric analysis of EdU^+ cardiomyocytes (% of cardiac-specific troponin T positive cells (TnT^+)) treated with naïve ExoCPC, Exo from CPC transfected with a siRNA against POSTN (ExoCPC_SiPOSTN), or PBS (Ctrl; *n* = 3; ** *p=0.0019; *** p=0.0002*). **F** Flow cytometry analysis of EdU^+ hiPS cell-derived cardiomyocytes (% of $cTnT^+$ cells) treated with naïve ExoCPC, ExoCPC_SiPOSTN, or PBS (Ctrl; *n* = 3; ** *p=0.0021* and *p=0.0045*).

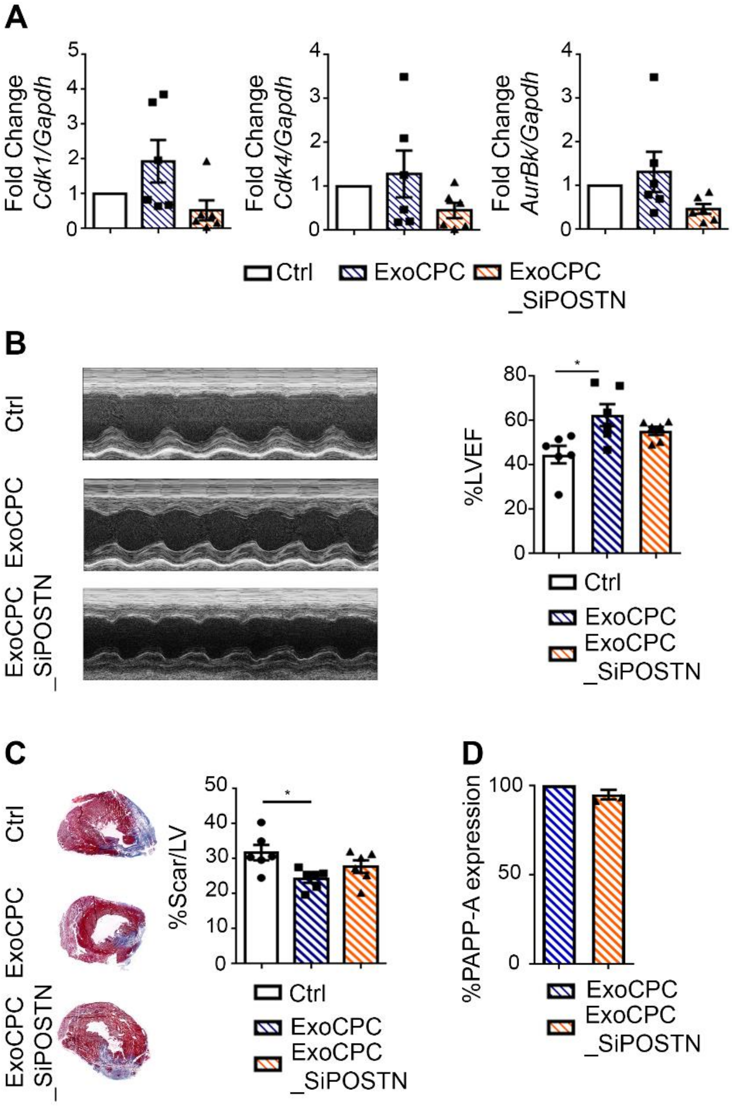

Figure S6: Effects of CPC-derived exosomes in an *in vivo* model of adult rat myocardial infarction (MI).

A Real-time RT-PCR analysis of *Cdk1*, *Cdk4*, and *AurBk* mRNA expression in dispersed isolated cardiomyocytes from rat hearts at day 14 post-MI. Infarcted rat hearts were injected intramyocardially with naïve ExoCPC, Exo from CPC transfected with a siRNA against periostin (ExoCPC_SiPOSTN), or PBS (Ctrl; 6 animals/group). **B** Left panel: Representative M-mode echocardiographs at day 14 post-MI in the different groups. Right panel: Quantitative analysis of left ventricular ejection fraction (LVEF; %) at day 14 post-MI (*n* = 6; * *p=0.0124*). **C** Representative heart sections at day 14 post-MI stained with Masson-trichrome. Quantitative analysis of infarct scar size (% of LV area; *n* = 6; * *p=0.0251*). **D** Western blot analysis of pregnancy-associated plasma protein-A (PAPP-A) expression, normalized for SYNTENIN-1 expression, in naïve ExoCPC and ExoCPC_SiPOSTN. (% ExoCPC; *n* = 3).

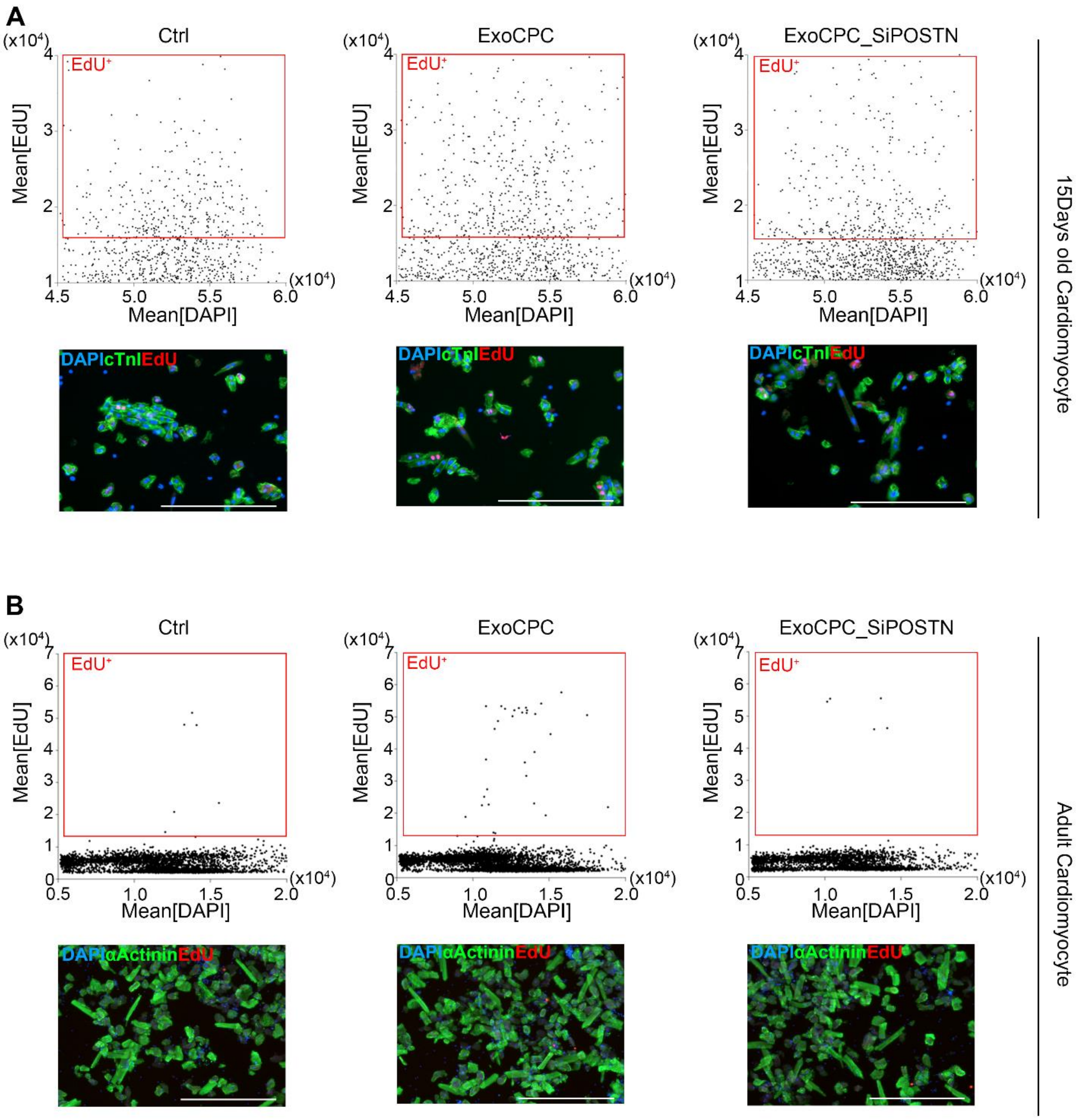

Figure S7: EdU nuclear incorporation analysis in dispersed isolated cardiomyocytes

A Dispersed isolated neonatal rat cardiomyocyte (see Figure 5E for the schematic protocol of the treatment) immunostained for EdU (red) and cardiac-specific troponin I (cTnI; green). Scale bar: 200µm. Representative scatter plot of EdU+ nuclei on DAPI stained nuclei are shown. Quantitative analysis is shown in Figure 5E. **B** Dispersed isolated adult rat cardiomyocyte from hearts explanted at day 14 post-MI immunostained for EdU (red) and cardiac-specific sarcomeric α-actinin (green).

Scale bar: 200µm. Representative scatter plot of EdU+ nuclei on DAPI stained nuclei are shown. Quantitative analysis is shown in Figure 6C.

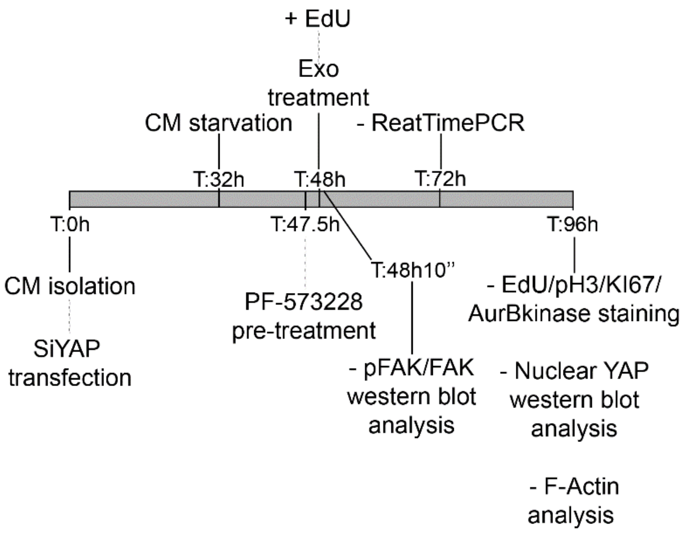

Figure S8: *In vitro* experimental timeline

Schematic of the experimental protocol of *in vitro* assays on neonatal rat cardiomyocytes. The relevant time points (T) are indicated.

Supplementary Tables:

Supplementary Table 1

Accession	Description	nPSM
**30102**	type I collagen [Homo sapiens]	185,6
**119590947**	fibronectin 1, isoform CRA_l [Homo sapiens]	184,2
**762938**	unnamed protein product [Homo sapiens]	130,3
**211904152**	glia-derived nexin isoform b precursor [Homo sapiens]	116,0
**378404908**	glyceraldehyde-3-phosphate dehydrogenase isoform 2 [Homo sapiens]	114,2
**557786190**	**periostin isoform 7 precursor [Homo sapiens]**	98,4

**119623487**	histone 1, H2bj, isoform CRA_b [Homo sapiens]	57,8
**194388798**	unnamed protein product [Homo sapiens]	44,6
**109150416**	peroxidasin homolog precursor [Homo sapiens]	44,2
**530366841**	PREDICTED: peroxidasin homolog isoform X1 [Homo sapiens]	42,5
**86651742**	tyrosine 3-monooxygenasea/tryptophan 5-monooxygenase activation protein zeta [Homo sapiens]	42,4
**119591511**	collagen, type VI, alpha 3, isoform CRA_c [Homo sapiens]	40,2
**8101724**	AF258350_1 canstatin, partial [Homo sapiens]	40,0
**133778299**	TUBB2B protein [Homo sapiens]	38,9
**158256710**	unnamed protein product [Homo sapiens]	37,9
**89243632**	ITGB1 protein [Homo sapiens]	35,7
**530400269**	PREDICTED: myosin light polypeptide 6 isoform X6 [Homo sapiens]	34,4
**87196339**	collagen alpha-1(VI) chain precursor [Homo sapiens]	32,3
**40226101**	ACTG1 protein, partial [Homo sapiens]	30,6
**386997**	prebeta-migrating plasminogen activator inhibitor, partial [Homo sapiens]	30,2
**193787599**	unnamed protein product [Homo sapiens]	28,7
**16741721**	Procollagen-lysine 1, 2-oxoglutarate 5-dioxygenase 1 [Homo sapiens]	27,5
**119625664**	annexin A5, isoform CRA_c [Homo sapiens]	24,3
**340234**	vimentin, partial [Homo sapiens]	22,8
**11935049**	AF304164_1 keratin 1 [Homo sapiens]	21,2
**221042224**	unnamed protein product [Homo sapiens]	21,2
**34069**	unnamed protein product [Homo sapiens]	20,4
**12667788**	myosin-9 [Homo sapiens]	19,0

**34782802**	PKM2 protein, partial [Homo sapiens]	18,8
**194376948**	unnamed protein product [Homo sapiens]	18,3
**148922238**	Thrombospondin 2 [Homo sapiens]	17,7
**119615401**	heparan sulfate proteoglycan 2 (perlecan), isoform CRA_b [Homo sapiens]	16,0
**282721077**	sushi repeat-containing protein SRPX isoform 3 precursor [Homo sapiens]	15,5
**119615036**	collagen, type I, alpha 1, isoform CRA_a [Homo sapiens]	15,4
**530425079**	PREDICTED: tubulin beta-4A chain isoform X2 [Homo sapiens]	14,8
**115527062**	collagen alpha-2(VI) chain isoform 2C2 precursor [Homo sapiens]	12,9
**84708830**	TUBB6 protein [Homo sapiens]	12,0
**119616320**	hyaluronan and proteoglycan link protein 1, isoform CRA_b [Homo sapiens]	11,8
**197692249**	annexin I [Homo sapiens]	10,3
**49257389**	CCDC80 protein [Homo sapiens]	10,3
**194384606**	unnamed protein product [Homo sapiens]	10,0
**38197240**	LAMB1 protein, partial [Homo sapiens]	8,4
**157653329**	procollagen C-endopeptidase enhancer 1 precursor [Homo sapiens]	8,3
**3777617**	serine protease [Homo sapiens]	8,2
**119616322**	EGF-like repeats and discoidin I-like domains 3, isoform CRA_a [Homo sapiens]	7,6
**12653033**	MYH10 protein [Homo sapiens]	7,3
**62089314**	Pro-alpha-1 type V collagen variant [Homo sapiens]	7,3
**189054446**	unnamed protein product [Homo sapiens]	6,3

**194097352**	alpha-actinin-1 isoform c [Homo sapiens]	5,8
**12653633**	Lysyl oxidase-like 2 [Homo sapiens]	5,8
**119582898**	MAM domain containing 2, isoform CRA_b [Homo sapiens]	5,6
**119590446**	nidogen 1, isoform CRA_b [Homo sapiens]	4,6
**189054446**	unnamed protein product [Homo sapiens]	3,8
**119607847**	pregnancy-associated plasma protein A, pappalysin 1, isoform CRA_c [Homo sapiens]	3,6
**119568019**	fibronectin type III domain containing 1 [Homo sapiens]	3,5
**30353925**	CLTC protein [Homo sapiens]	3,2
**9309503**	AC013451_1 LTBP-2 [Homo sapiens]	2,9
**553348**	hexabrachion, partial [Homo sapiens]	2,8
**119593150**	filamin A, alpha (actin binding protein 280), isoform CRA_a [Homo sapiens]	2,4
**118572606**	hemicentin-1 precursor [Homo sapiens]	1,0

Table S1: **Proteomic Analysis.**

Supplementary Table 2

		**Ctrl**		**ExoCPC**		**ExoCPC_SiPOSTN**	
		**Mean**	**SEM**	**Mean**	**SEM**	**Mean**	**SEM**
**CO**	**mL/min**	75,80	9,21	112,61	19,59	90,7	10,89
**EF**	**%**	44,69	3,92	62,50	4,95	55,41	1,842
**FAC**	**%**	35,20	7,08	54,93	3,87	47,85	4,69
**FS**	**%**	10,91	1,97	19,13	2,06	16,39	2,44
**SV**	**µL**	242,41	22,35	310,81	49,52	258,48	27,50
**Vd**	**µL**	560,32	62,15	484,93	40,87	462,79	38,66
**Vs**	**µL**	317,91	58,30	174,12	16,00	207,31	12,84

Table S2: Echocardiographic Data at Day 14 post-MI

CO: Cardiac Output; EF: Ejection Fraction; FAC: Fractional Area Change; FS: Fractional Shortening; SV: Stroke Volume; Vd: Diastolic Volume; Vs: Systolic Volume.

Supplementary Table 3

Antibody	Application	Dilution	Company	# Ref
**TSG101**	WB	1:1000	Abcam	ab125011
**SYNTENIN-1**	WB	1:1000	Abcam	ab133267
**CALNEXIN**	WB	1:300	SantaCruz Biotechnology	sc-70481
**POSTN**	WB	1:300	SantaCruz Biotechnology	sc-398631
**cTnI**	IF	1:200	Abcam	ab47003
**αSarcomeric**	IF	1:300	Abcam	ab9465
**pH3**	IF	1:300	Abcam	ab14955
**pAKT**	WB	1:1000	Cell Signaling	4060
**AKT**	WB	1:1000	Cell Signaling	9272
**GAPDH**	WB	1:5000	Abcam	ab181602
**CYCLIN D1**	WB	1:30000	Abcam	ab134175
**pFAK**	WB	1:1000	Abcam	ab81298
**FAK**	WB	1:1000	Abcam	ab76496
**YAP1**	WB	1:500	Invitrogen	PA5-87568
**H3**	WB	1:5000	Abcam	ab176842
**AuroraBKinase**	IF	1:250	BD	611083
**cTnT**	IF	1:100	Miltenyi Biotec	130-119-674

**CD63**	IF	1:100	SantaCruz Biotechnology	sc-365604
**PAPP-A**	WB	1:300	HyTest Ltd	4P41

Table S3: **Detailed information on antibodies used in the study.**
